# Global Profiling of Phosphorylation Reveals the Barley Roots Response to Phosphorus Starvation and Resupply

**DOI:** 10.3389/fpls.2021.676432

**Published:** 2021-07-14

**Authors:** Zengke Ma, Juncheng Wang, Chengdao Li, Panrong Ren, Lirong Yao, Baochun Li, Yaxiong Meng, Xiaole Ma, Erjing Si, Ke Yang, Xunwu Shang, Huajun Wang

**Affiliations:** ^1^Gansu Provincial Key Lab of Aridland Crop Science/Gansu Key Lab of Crop Improvement and Germplasm Enhancement, Lanzhou, China; ^2^Department of Crop Genetics and Breeding, College of Agronomy, Gansu Agricultural University, Lanzhou, China; ^3^Western Barley Genetics Alliance, College of Science, Health, Engineering and Education, Murdoch University, Murdoch, WA, Australia; ^4^Department of Botany, College of Life Sciences and Technology, Gansu Agricultural University, Lanzhou, China

**Keywords:** barley (*Hordeum vulgare*), phosphorylation, PTM, phosphorus starvation/resupply, metabolism

## Abstract

Phosphorus (P) deficiency is a major threat to the crop production, and for understanding the response mechanism of plant roots, P stress may facilitate the development of crops with increased tolerance. Phosphorylation plays a critical role in the regulation of proteins for plant responses to biotic and abiotic stress; however, its functions in P starvation/resupply are largely unknown for barley (*Hordeum vulgare*) growth. Here, we performed a global review of phosphorylation in barley roots treated by P starvation/resupply. We identified 7,710 phosphorylation sites on 3,373 proteins, of which 76 types of conserved motifs were extracted from 10,428 phosphorylated peptides. Most phosphorylated proteins were located in the nucleus (36%) and chloroplast (32%). Compared with the control, 186 and 131 phosphorylated proteins under P starvation condition and 156 and 111 phosphorylated proteins under P resupply condition showed significant differences at 6 and 48 h, respectively. These proteins mainly participated in carbohydrate metabolism, phytohormones, signal transduction, cell wall stress, and oxidases stress. Moreover, the pathways of the ribosome, RNA binding, protein transport, and metal binding were significantly enriched under P starvation, and only two pathways of ribosome and RNA binding were greatly enriched under Pi resupply according to the protein–protein interaction analysis. The results suggested that the phosphorylation proteins might play important roles in the metabolic processes of barley roots in response to Pi deficiency/resupply. The data not only provide unique access to phosphorylation reprogramming of plant roots under deficiency/resupply but also demonstrate the close cooperation between these phosphorylation proteins and key metabolic functions.

## Introduction

Phosphorus (P) is an essential macronutrient for plant growth, development, metabolism, and regulatory processes (Lopez-Arredondo et al., 2014; Cong et al., 2020). However, inorganic phosphate (Pi), the only form of P that can be acquired by plant roots, is not readily taken up by roots because of its low diffusion rate and instability of forms (Wu et al., 2013). Globally, almost 30% of the cultivated land experiences Pi deficiency (Macdonald et al., 2011). Therefore, a lot of Pi fertilizer is applied to low-Pi soils to increase crop yield; however, crops can acquire, at most 30% of applied Pi, which has led to the excess application of Pi fertilizers with the phenomenon of eutrophication of surface waters, and soil hardening (Correl, 1998; Macdonald et al., 2011; Lopez-Arredondo et al., 2014). In addition, increasing consumption of Pi fertilizer exacerbates the depletion of non-renewable rock phosphate (Johnston et al., 2014). Thus, it is critical to understand the mechanism of Pi homeostasis and to screen Pi-efficient crop genotypes for promoting Pi-use efficiency.

In order to cope with low-Pi conditions, plants have evolved a range of elaborate strategies at the morphological, physiological, and molecular levels to enhance their acquisition efficiency and utilization efficiency of Pi (Yang et al., 2019). Especially, these strategies are closely related to root traits including architectural, morphological, physiological, and symbiotic traits (Niu et al., 2012; Sawers et al., 2017; Kafle et al., 2019). In addition, high- and low-affinity Pi transporters are involved in Pi uptake and transport activities (Parra-Almuna et al., 2020). For example, the best understood, phosphate transporter 1 (PHT1) is mainly expressed in roots and is involved in the absorption, distribution, and remobilization of phosphate (Zhang et al., 2019a; Parra-Almuna et al., 2020). In addition, changes of root system architecture involved in plant response to P deficiency are regulated by various factors, including phytohormones, sucrose, and transcriptional modification (Chiou and Lin, 2010; Dai et al., 2016). Transcriptomic, proteomic, and metabolomic platforms have been applied to characterize the Pi stress response (Sha et al., 2016; Luo et al., 2018; Ren et al., 2018). A proteomic study showed that many differentially accumulated proteins took part in carbon and energy metabolism, signal transduction, secondary metabolism, and stress defense associated with low-Pi tolerance in barley (Nadira et al., 2016). Recently, posttranscriptional modification was shown to play a key role in the plant response to P deficiency (Park et al., 2014; Yang et al., 2019, 2020; Wang et al., 2021). *Arabidopsis thaliana* PHOSPHATE2 (AtPHO2) and NITROGEN LIMITATION ADAPTATION (AtNLA) as E2 conjugase and E3 ubiquitin ligase enzymes, respectively, were found to degrade several PHT1 members (Huang et al., 2013; Lin et al., 2013; Park et al., 2014). In rice, decreased phosphorylation levels of OsMAPK6, OsCK2, and five calcium-dependent protein kinases (CDPKs) were found in response to phosphate starvation (Yang et al., 2019). The OsPP95, a protein phosphatase type 2C (PP2C), regulates dephosphorylation and transportation of phosphate transporters to maintain P homeostasis in rice (Yang et al., 2020).

Protein phosphorylation is widely used to study posttranslational modifications (PTMs) and mainly effects the hydroxyl groups of threonine, serine, and tyrosine in eukaryotic cells (Reinders and Sickmann, 2005). In order to understand the regulatory mechanisms of the Pi tolerance, numerous phosphorylation proteins have been identified in many plants, including *A. thaliana*, rice, and wheat (Zhang et al., 2016; Yang et al., 2019). Barley (*Hordeum vulgare* L.) is one of the oldest cereal crops and is ranked fourth in the world cereal production. It is widely used as a major food source, livestock feed, and raw material for malting and brewing (FAO., 2018) and displays wide adaptation to extreme environments. After many years of high-nutrient breeding, crop varieties with good tolerance to limited nutrients are often eliminated, and most research on P is derived from model species, not crops (Oldroyd and Leyser, 2020). Our previous efforts to identify two barley lines with different P efficiency were screened from cultivated barley (Ren et al., 2016), but there is little underlying knowledge of phosphorylation modification under Pi deficiency/resupply in barley.

In this study, a high P-efficiency barley genotype GN121 was used for a comprehensive phosphorylation analysis under Pi deficiency/resupply. We adopted high-resolution liquid chromatography tandem mass spectrometry (LC-MS/MS) linked to highly sensitive immune-affinity antibody analysis and powerful bioinformatics, which were used to identify the substrates and describe the functional characteristics to explore the interplay of phosphorylation in barley roots. To the best of our knowledge, this is the first comprehensive analysis of phosphorylation in barley roots. Our research not only greatly extends the list of phosphorylated proteins in barley but also widens the knowledge of P stress response and defense mechanisms in crops.

## Materials and Methods

### Barley Materials and Growth Conditions

The low-Pi-tolerant barley GN121, which reportedly has strong resistance to low Pi stress, was used for phosphoproteomics work (Ren et al., 2018). The nutrient solution and growth conditions of the hydroponic culture are described by Ren et al. (2018). Briefly, surface-sterilized seeds were germinated in a Petri dish on a double-layered filter paper and grown in a greenhouse for 10 days, and then transferred to modified Hoagland hydroponic nutrient solution containing 0.397 mM KH_2_PO_4_ (high Pi; +Pi) or 0.0397 mM KH_2_PO_4_ (low Pi; –Pi) Pi concentrations. Seedlings (10 days old) were subjected to low Pi for 48 h and then resupplied with high Pi for 48 h. The roots were sampled after Pi starvation for 6 h (P6) and 48 h (P48), and 6 h (R6) and 48 h (R48) of Pi resupply conditions, the roots before treatment were used as control (CK). All roots were directly harvested into liquid nitrogen and stored at −80° for subsequent protein extraction.

### Protein Extraction

The samples were fully ground to powder in liquid nitrogen before being transferred to a 5-ml centrifuge tube. Then, 2 ml lysis buffer [8 M urea, 1% Triton-100, 10 mM dithiothreitol (DTT), and 1% Protease Inhibitor Cocktail (Calbiochem, Darmstadt, Germany)] were added to the powder, and the mixtures were sonicated three times on ice using a high-intensity ultrasonic processor. The remaining debris was removed by centrifugation at 20,000× *g* for 10 min at 4°C. The proteins were precipitated with cold 20% trichloroacetic acid for 2 h at −20°C. After centrifugation at 12,000× *g* for 10 min at 4°C, the supernatant was discarded. The remaining precipitate was washed three times with cold acetone. The protein was redissolved in 8 M urea, and the protein concentration was determined with a Pierce BCA Protein Assay Kit (Pierce, Thermo Fisher Scientific, Bonn, Germany) kit according to the instructions of the manufacturer.

### Trypsin Digestion

The procedures were as described by Zhong et al. (2017). Dithiothreitol was added to the protein solution to a final concentration of 5 mM and reduced at 56°C for 30 min. Then, this mixture was alkylated with iodoacetamide to 11 mM for 15 min at room temperature in darkness. The treated protein samples were then diluted by adding 100 mM triethylammonium bicarbonate (TEAB, Sigma, Germany) to urea at <2 M concentration. Finally, trypsin was added at 1:50 trypsin-to-protein mass ratio for the first digestion overnight and 1:100 trypsin-to-protein mass ratio for a second 4-h digestion.

### Enrichment for Phosphorylated Peptides

To enrich modified peptides, tryptic peptides dissolved in the NETN buffer (100 mM NaCl, 1 mM EDTA, 50 mM Tris–HCl, 0.5% NP-40 and pH 8.0) were incubated with prewashed antibody beads (Lot number 001, PTM BIOLABS, Chicago, IL, USA) at 4°C overnight with gentle shaking. The beads were washed four times with the NETN buffer and twice with ddH_2_O. The bound peptides were eluted from the beads with 0.1% trifluoroacetic acid. Finally, the eluted fractions were combined and vacuum-dried. The resulting peptides were desalted with C18 Zip Tips (Millipore, Darmstadt, Germany) according to the instructions of the manufacturer for the LC-MS/MS analysis.

### The LC-MS/MS Analysis

The tryptic peptides were dissolved in 0.1% formic acid (solvent A), directly loaded onto a home-made reversed-phase analytical column (15 cm length, 75 μm i.d.). The gradient comprised an increase from 6 to 23% solvent B (0.1% formic acid in 98% acetonitrile) over 26 min, 23–35% in 8 min, climbing to 80% in 3 min, and then holding at 80% for the last 3 min, all at a constant flow rate of 400 nl/min on an EASY-nLC 1000 UPLC System (Bruker Daltonics).

The peptides were subjected to nanospray ionization (NSI) source followed by tandem mass spectrometry (MS/MS) in Q Exactive^*TM*^ Plus (Thermo Fisher Scientific, Bremen, Germany) coupled online to the UPLC system. The electrospray voltage applied was 2 kV. The *m*/*z* scan range was 350–1,800 for a full scan, and intact peptides were detected in the Orbitrap at a resolution of 70,000. Peptides were then selected for MS/MS using normalized collision energy (NCE) setting as of 28, and the fragments were detected in the Orbitrap at a resolution of 17,500. A data-dependent procedure that alternated between one MS scan followed by 20 MS/MS scans with 15.0 s dynamic exclusion. Automatic gain control (AGC) was set at 5E4. Fixed first mass was set as 100 *m*/*z*.

### Database Search

The resulting MS/MS data were processed using the Maxquant search engine (v.1.6.6.0; Max Plank Institute of Biochemistry, Germany). The MS/MS spectra were searched against the human UniProt database (http://www.ebi.ac.uk/GOA/) concatenated with reverse decoy database. Trypsin/P was specified as a cleavage enzyme allowing up to four missing cleavages. The mass tolerance for precursor ions was set as 20 ppm in the First search and 5 ppm in the Main search, and the mass tolerance for fragment ions was set as 0.02 Da. Carbamidom108ethyl on Cys was specified as fixed modification, and acetylation modification and oxidation on Met were specified as variable modifications. The false discover rate was adjusted to <1%, and the minimum score for modified peptides was set >40. A label-free quantification (LFQ) method was based on extracted ion currents (XICs) and was used to quantify phosphorylated protein abundance.

### Bioinformatics Annotation Analysis

Gene Ontology (GO) annotation proteome was based on three categories (biological process, cellular component, and molecular function) and derived from the UniProt-GOA database (http://www.ebi.ac.uk/GOA/). Identified protein domain functional description was annotated by InterProScan (http://www.ebi.ac.uk/interpro/) based on the protein sequence alignment method. Eukaryotes database of Wolfpsort (http://www.genscript.com/psort/wolf_psort.html) was used to identify the subcellular localization of proteins. Soft MoMo (http://meme-suite.org/tools/momo), a motif-x algorithm, was used to analyze the model of sequences constituted with amino acids in specific positions of modify-13-mers (six amino acids upstream and downstream of the site) in all protein sequences. STRING database version 10.1 (https://www.string-db.org/) was deployed to analyze the protein–protein interaction (PPI) of the phosphoproteins identified in the current study, applying a confidence score of 0.7 (high confidence).

## Results

### Identification of Phosphorylated Sites and Proteins in Barley Roots

**Figure 1:**
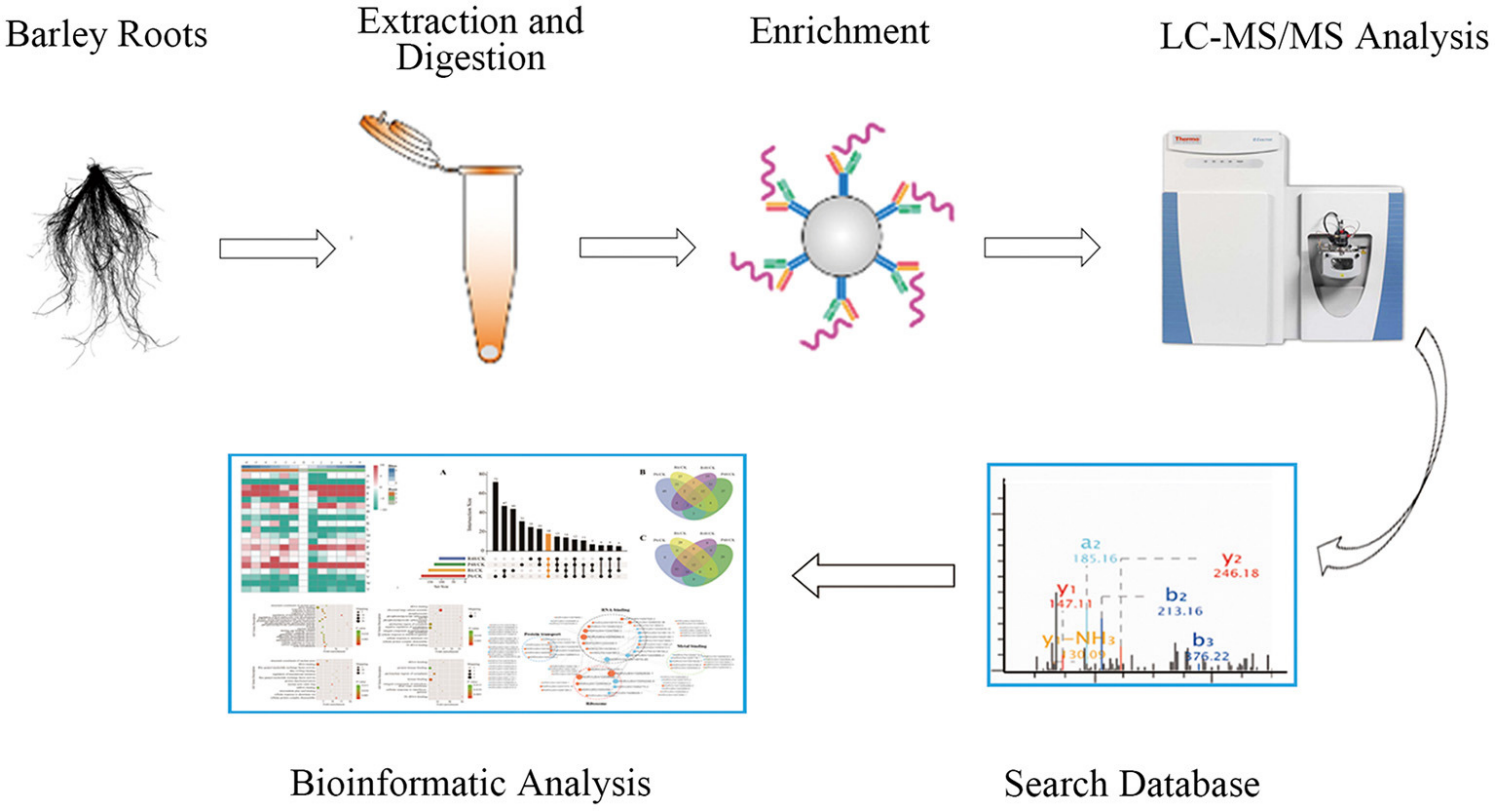
The workflow of integrated strategy for global mapping of phosphorylation in barley roots.

**Figure 2:**
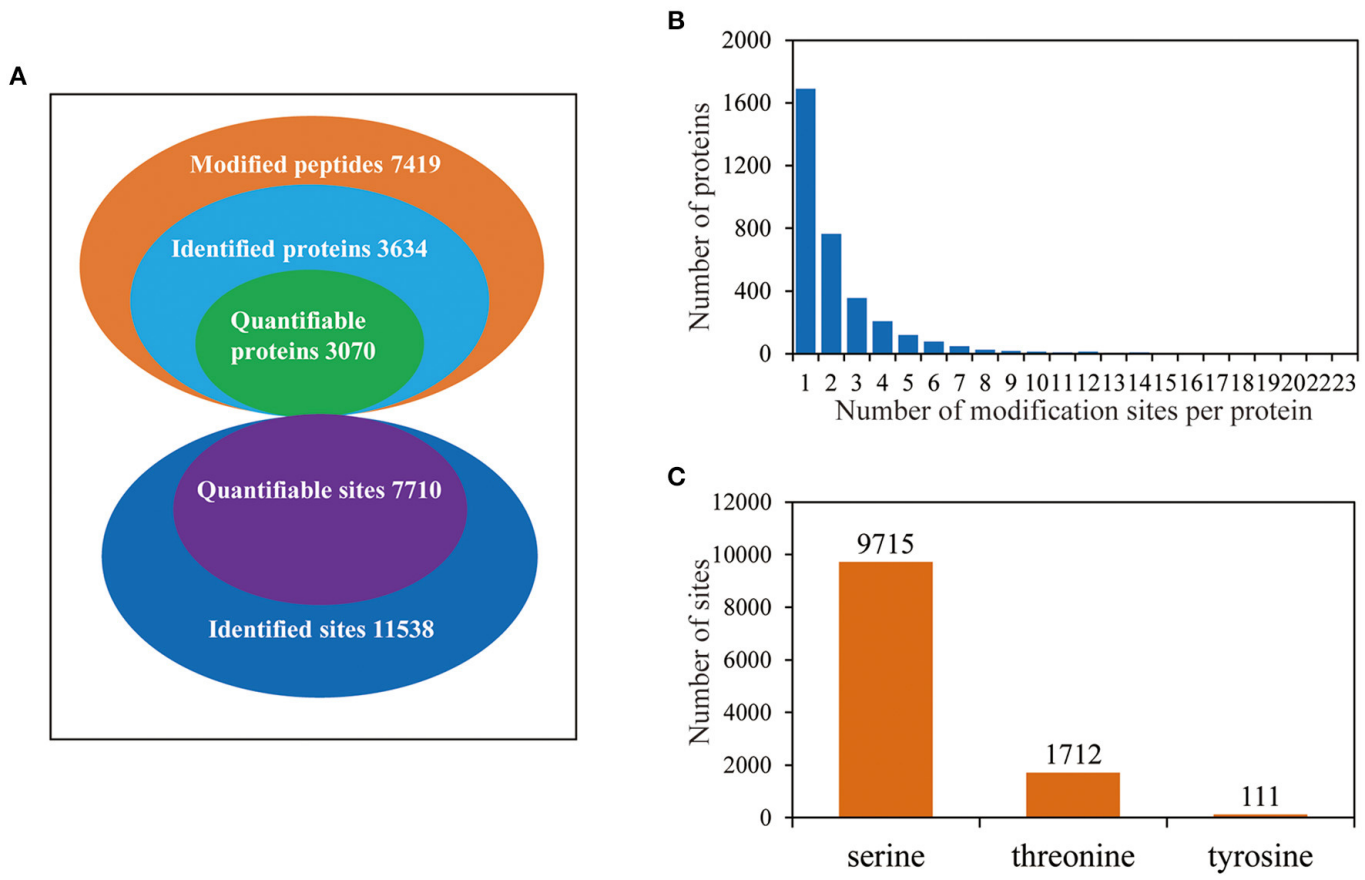
The proteome-wide identification of phosphorylation proteins and sites in barley roots. **(A)** Summary of the phosphorylated proteins and sites identified and quantified; **(B)** peptide length distributions of phosphorylation profiles; **(C)** distribution patterns of phosphorylation occurring on serine, threonine, and tyrosine.

To investigate the phosphorylation profiling of barley (GN121) roots under Pi deficiency/resupply, a combination of iTRAQ-based quantitative proteomic and LC–MS/MS method was used to identify phosphorylated proteins and phosphorylation sites (Figure 1). The mass spectrometry data have been deposited at the ProteomeXchange (http://proteomecentral.proteomexchange.org/cgi/GetDataset) with dataset identifier PXD022053 and PXD022077. A total of 7,710 phosphorylation sites were identified, associated with 3,070 proteins that were quantified (Figure 2A and Supplementary Table 1). The results indicated that 50.1% contained a single phosphorylated site, 22.7% contained two phosphorylated sites, 10.6% contained three phosphorylated sites, and 6.1% contained four phosphorylated sites. Additionally, the phosphorylated sites of 50 (1.48%) proteins were above 10, and there were at least 20 phosphorylated sites in four proteins (Figure 2B). In addition, we counted the phosphorylation sites on serine, threonine, and tyrosine. A total of 11,538 phosphorylation sites were detected, with 9,715 on serine (84.2%), 1,712 on threonine (14.8%), and 111 on tyrosine (0.9%) sites (Figure 2C). The distribution patterns of phosphorylation types were consistent with other previous reports on wheat and rice (Zhang et al., 2014a; Zhong et al., 2017).

### Motif Analysis of Phosphorylation Sites

**Figure 3:**
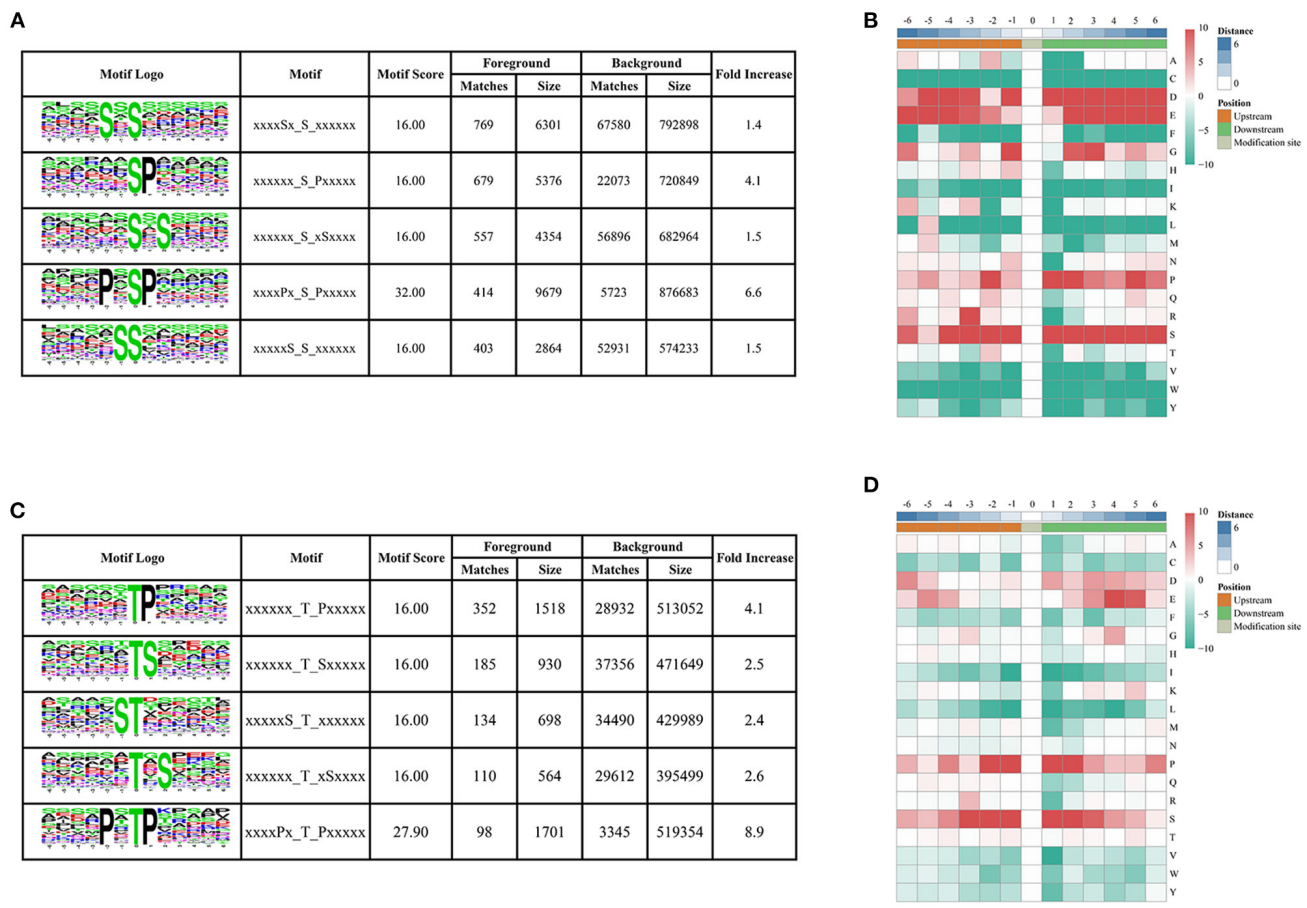
Motif analysis of phosphorylated peptides. **(A,C)** Top five serine and threonine phosphorylation motifs; **(B,D)** sequence probability logos of significantly enriched phosphorylation site motifs for six amino acids around the serine and threonine phosphorylation sites.

To determine the conserved motifs surrounding the phosphorylation sites, the MoMo software (http://meme-suite.org/tools/momo) was employed to identify the kinase-associated phosphorylation motifs of the phosphoproteins. We extracted a 13 amino-acid sequence centered on the phosphorylation site and obtained 10,428 distinct sequences, including 8,978 phosphoserines, 1,428 phosphothreonines, and 22 phosphotyrosines (Supplementary Table 2). Moreover, 61, 14, and one types of conserved motifs were significantly enriched around the phosphoserine, phosphothreonine, and phosphotyrosine sites, respectively (Figure 3 and Supplementary Table 2). The most common motifs were “Sxs” with 769 matches, followed by “sP” (679) and “sxS” (557). There were also more than 400 occurrences each of “PxsP,” and “Ss,” and over 200 occurrences each of “tP,” “sxE,” “RSxs,” “sSP,” “Gs,” “sPxxS,” “sxD,” “sxSP,” “Sxxs,” and “Rxxs” motifs, and also other motifs (Supplementary Figure 1). The “sP” has been reported to be substrate of mitogen-activated protein kinases (MAPKs), sucrose non-fermenting1-related protein kinase 2 (SnRK2s), AGC (cAMP- and cGMP-dependent protein kinase C), and many other kinases (Wijk et al., 2014; Zhang et al., 2014b). We also identified “Rxxs” and “LxRxxs” motifs that could be recognized by MAPKK, CaMK (calmodulin-dependent protein kinase)-II, and protein kinase A. In plants, the “tP” proved to be the most common phosphothreonine motif (Wijk et al., 2014), corresponding to MAPK substrates (Yang et al., 2019). Similarly, the motif “tP” was the most commonly present around phosphothreonine motifs with 352 occurrences; “Dy” was the only conserved phosphotyrosine motif in this research.

### Functional Characterization and Subcellular Localization of Phosphorylated Proteins in Barley Roots

**Figure 4:**
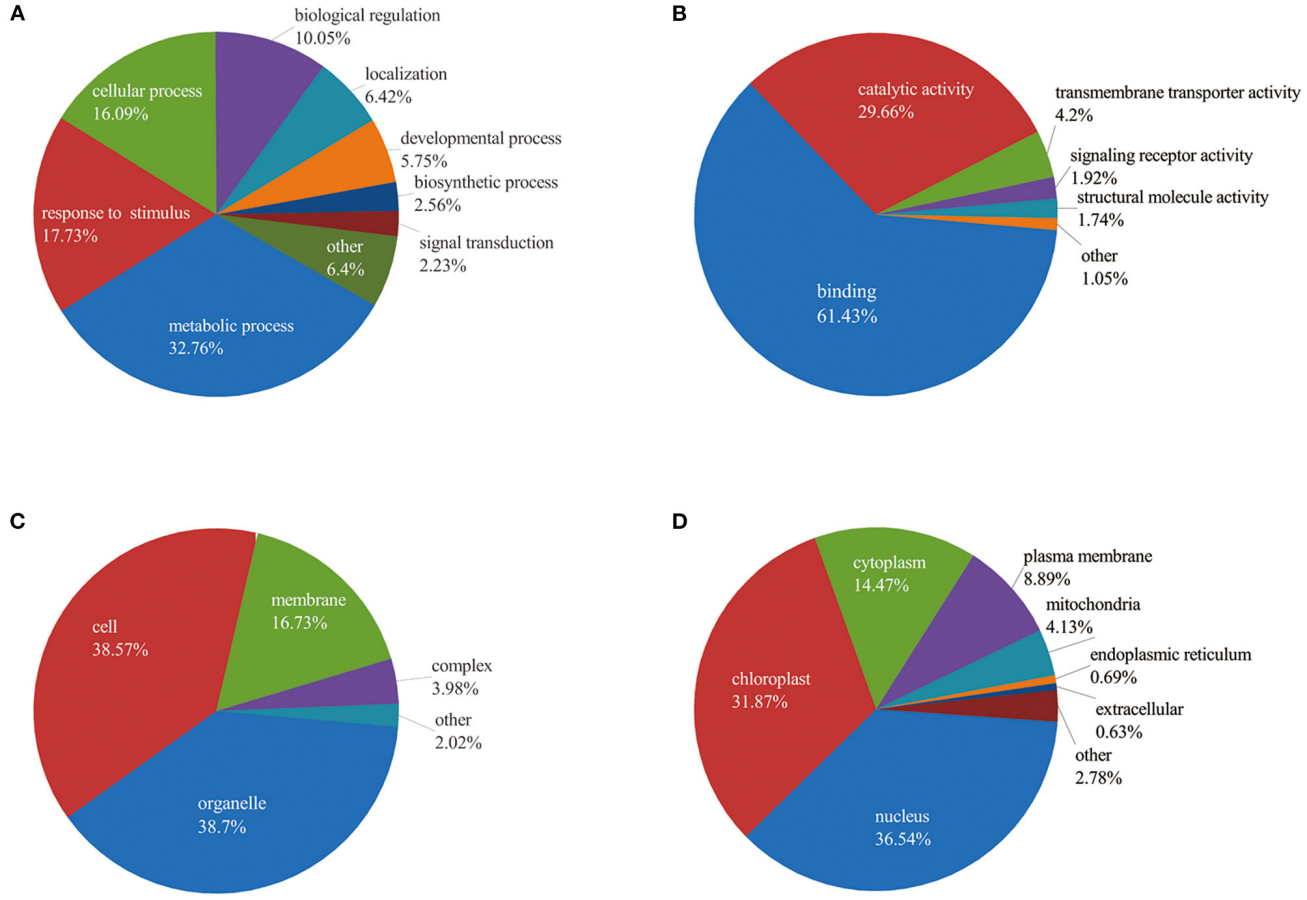
Functional classification of phosphorylated proteins in barley roots based on: **(A)** biological process; **(B)** molecular function; **(C)** cellular component; and **(D)** subcellular localization of phosphorylated proteins.

To better understand the potential roles of phosphorylation in barley roots, the GO analysis was used to assign them to biological process, molecular function, and cellular component (Figure 4 and Supplementary Table 3). In the biological process, most phosphorylated proteins were involved in metabolic processes (33%), response to stimulus (18%), and cellular processes (16%) (Figure 4A). The most common molecular functions were binding (61%) and catalytic activity (30%) (Figure 4B). For the cellular component, a majority of phosphorylated proteins were related to organelle (39%), cell (38%), and membrane (17%) (Figure 4C).

Furthermore, we explored the biological process of phosphorylated proteins involved in response to stimulus and signal transduction. In detail, proteins involved in the stimulus response included calmodulin-binding transcription activator 2 (HORVU4Hr1G078620.5), mitogen-activated protein kinase 1 (MAPK1, HORVU7Hr1G097740.1), polyol/monosaccharide transporter 5 (HORVU1Hr1G052040.1), and some protein kinase family proteins. Signal transduction process included MAPK1, CDPK 19 (HORVU5Hr1G110900.3), ethylene-insensitive protein 2 (EIN2, HORVU5Hr1G050330.2), abscisic acid (ABA) receptor PYR1 (PYR1, HORVU3Hr1G031380.1), and protein kinase. In addition, subcellular localization showed that proteins were mainly located in nucleus (36%), chloroplast (32%), and cytoplasm (14%). Several proteins were found in the plasma membrane (9%), mitochondria (4%), endoplasmic reticulum (13%), and extracellular (1%) (Figure 4D). These results indicate that the phosphorylated proteins are located in different cellular compartments and involved in numerous biological processes in barley.

### Design of Venn Diagram and Functional Analysis of Phosphorylated Proteins

**Figure 5:**
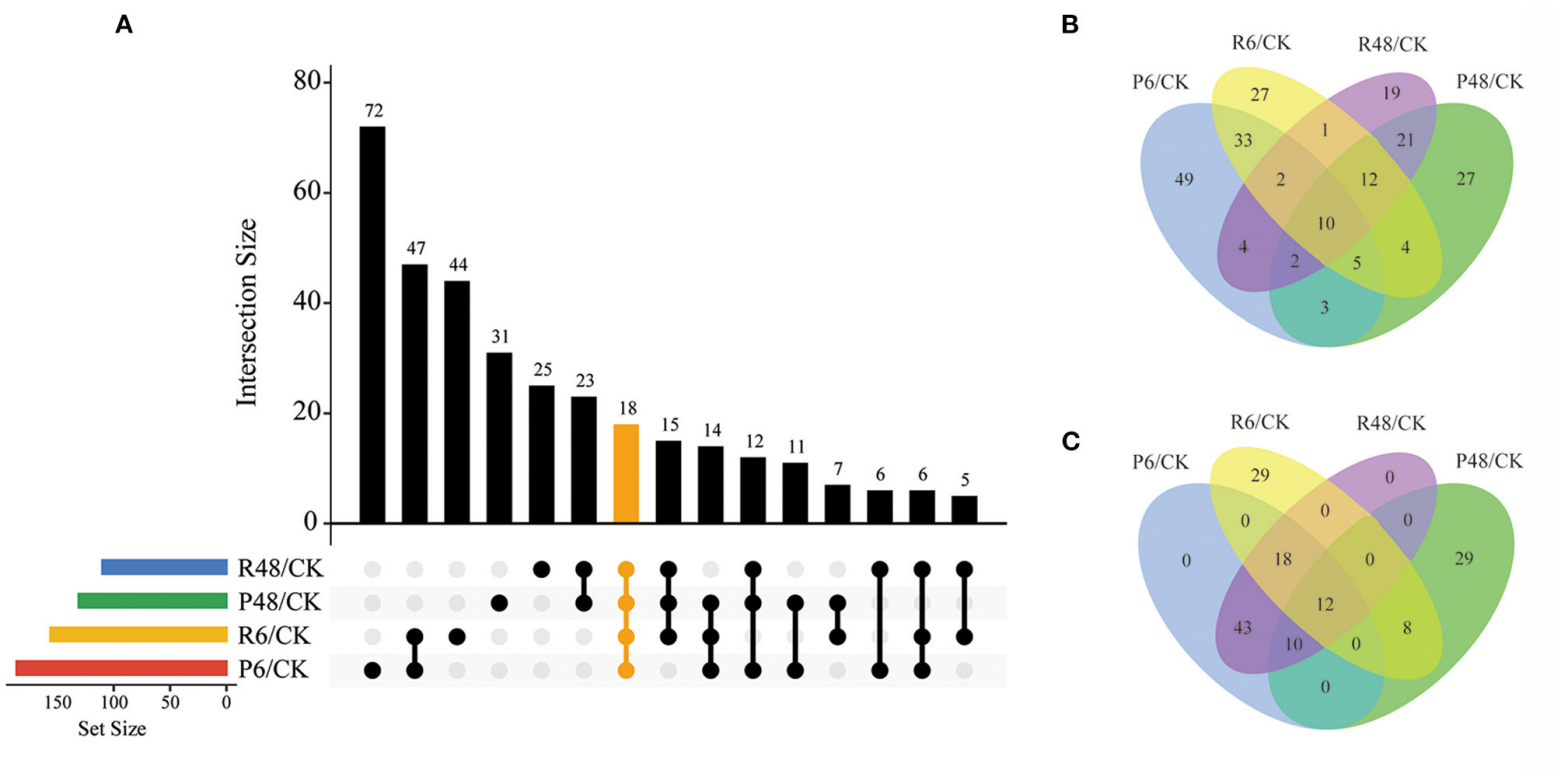
Venn diagram analysis of DPPs under different treatments. **(A)** All DPPs for the four treatments. The bar charts indicate the number of DPPs under Pi starvation for 6 h (P6/CK) and 48 h (P48/CK), and Pi resupply 6 h (R6/CK) and 48 h (R48/CK); **(B)** Upregulated DPPs identified in P6/CK, P48/CK, R6/CK, and R48/CK; **(C)** downregulated DPPs identified in P6/CK, P48/CK, R6/CK, and R48/CK. DPPs, differentially phosphorylated proteins.

The change of phosphorylation pattern can be used to explore the potential function of phosphorylation in biological processes (Hou et al., 2017). We identified differentially phosphorylated proteins (DPPs) and sites by at least 1.5 quantification ratio change or more (*p* < 0.05) within each treatment and constructed a Venn diagram based on DPPs (Figure 5 and Supplementary Table 4).

In total, we identified 327 DPPs, of which 209 were upregulated and 149 were downregulated in barley roots. In detail, for DPPs upregulated compared with the control, 49, 17, 27, and 19 DPPs were specifically observed at P6, P48, R6, and R48, respectively; and 10 DPPs overlapped with four groups. Consistently, for downregulated DPPs, 29 were only observed at P48 and R6, respectively, and 12 overlapped with four groups. Many of these overlapping proteins were involved in different biological processes. For example, AMP deaminase (HORVU1Hr1G059810.2 and HORVU2Hr1G014130.4) participated in purine metabolism; a protein kinase superfamily protein (HORVU7Hr1G055980.7) participated in signal transduction; and polyol/monosaccharide transporter 5 (HORVU1Hr1G052040.1), trehalose phosphate synthase (TPS, HORVU1Hr1G076480.3), and phosphoenolpyruvate carboxylase 3 (PEPC3, HORVU7Hr1G080510.2) participated in carbohydrate metabolism.

**Figure 6:**
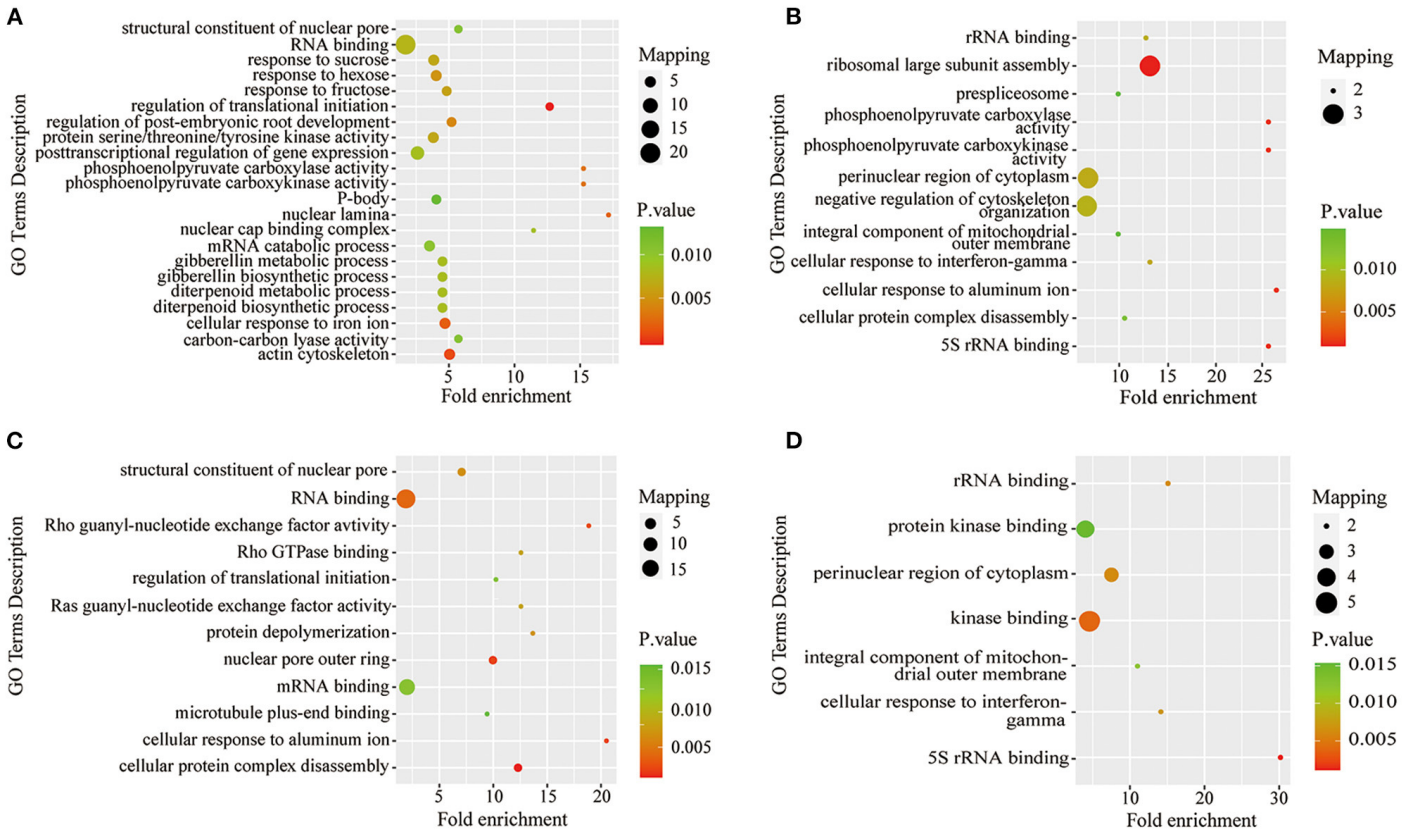
GO enrichment analysis of DPPs in roots under Pi starvation for 6 and 48 h, and Pi resupply for 6 and 48 h. DPPs, differentially phosphorylated proteins. **(A)** GO enrichment analysis of P6/CK; **(B)** GO enrichment analysis of P48/CK; **(C)** GO enrichment analysis off R6/CK; **(D)** GO enrichment analysis of R48/CK.

**Figure 7:**
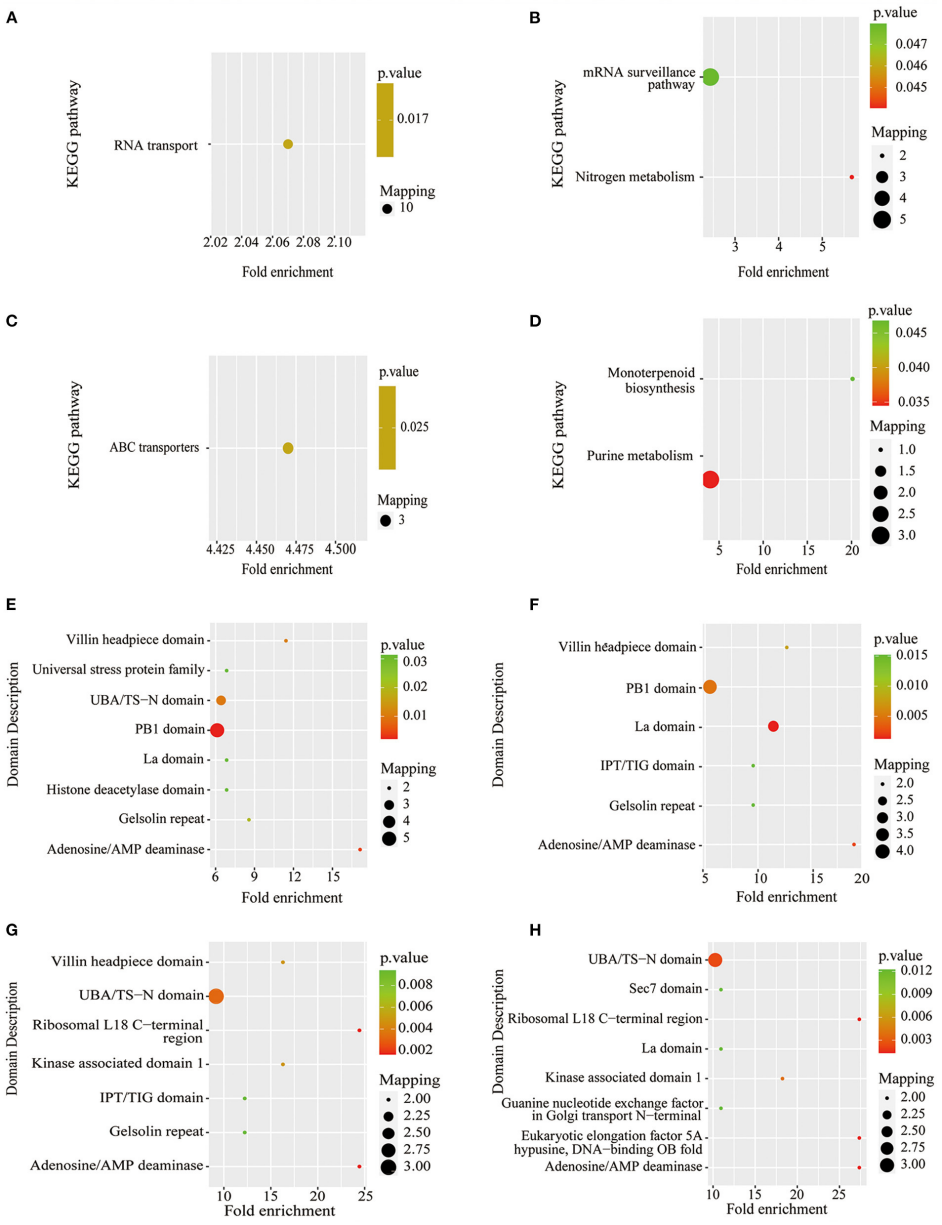
KEGG pathway-based enrichment analysis and protein domain enrichment analysis of Pi starvation and resupply. **(A)** KEGG pathway-based enrichment analysis of P6/CK; **(B)** KEGG pathway-based enrichment analysis of P48/CK; **(C)** protein domain enrichment analysis of R6/CK; **(D)** KEGG pathway-based enrichment analysis of R48/CK; **(E)** protein domain enrichment analysis of P6/CK; **(F)** protein domain enrichment analysis of P48/CK; **(G)** protein domain enrichment analysis of R6/CK; **(H)** protein domain enrichment analysis of R48/CK. KEGG, Kyoto Encyclopedia of Genes and Genomes.

To further understand the characteristics of DPPs, analyses of GO (biological process, molecular function, and cellular component), Kyoto Encyclopedia of Genes and Genomes (KEGG) pathway, and protein domains were performed (Figures 6, 7 and Supplementary Tables 5–7). The results showed that the GO terms of DPPs were significantly enriched at P6/CK, including regulation of translational initiation, cellular response to iron ion, PEPC activity, actin cytoskeleton, and nuclear lamina (Figure 6A). The GO terms of DPPs were significantly enriched at P48/CK, such as ribosomal large subunit assembly, cellular response to aluminum ion, rRNA binding, phosphoenolpyruvate carboxy kinase activity, and PEPC activity (Figure 6B). Consistently, for the P resupply treatment at R6/CK, the GO terms were mainly enriched in cellular protein complex disassembly, cellular response to aluminum ion, structural constituent of the nuclear pore, nuclear pore outer ring, and RNA binding (Figure 6C). For R48/CK, the GO terms mainly enriched were 5S rRNA binding, cellular response to interferon-gamma, kinase binding, rRNA binding, and perinuclear region of cytoplasm (Figure 6D).

The KEGG pathway analysis showed that the protein synthesis and degradation (RNA transport, mRNA surveillance pathway, and ABC transporters) were significantly enriched for P6/CK, P48/CK, and R6/CK. Furthermore, the metabolic process (nitrogen and purine metabolism) and biosynthesis (monoterpenoid biosynthesis) were significantly enriched for P48/CK and R48/CK (Figures 7A–D). Domain enrichment analysis showed that the most common forms of phosphorylated proteins were the domains of adenosine/AMP deaminase, UBA/TS-N, PB1, Villin headpiece, and La (Figures 7E,F). Above all, these results proved that phosphorylated proteins widely participate in diverse pathways in response to the Pi change.

### Kinases and Phosphatase Proteins

Protein kinases and protein phosphatases are two vital antagonistic components involved in protein phosphorylation (Uhrig et al., 2013). In the present study, a total of 40 and 10 DPPs were identified as protein kinases and phosphatases under Pi starvation/resupply, respectively. In detail, under the Pi starvation treatment, 21 kinases and three phosphatases were identified at P6/CK, and 15 kinases and four phosphatases were identified at P48/CK. Consistently, under the Pi resupply treatment condition, 18 kinases and three phosphatases were identified at R6/CK, and 19 kinases and two phosphatases were identified at R48/CK. Interestingly, these protein kinases and protein phosphatases had characteristics of poly-phosphorylation sites; For example, a protein kinase superfamily protein (HORVU2Hr1G110900.9) showed significantly upregulated phosphorylation at a total of six sites under four different treatments, suggesting that it was a potential component of the Pi-signaling cascade. In addition, other enzymes, such as AMP deaminase (HORVU1Hr1G059810.2 and HORVU2Hr1G014130.4), DNA helicase MCM8 (HORVU1Hr1G063700.2), TPS (HORVU1Hr1G076480.3), pyridoxal 5′ phosphate synthase subunit PdxS (HORVU2Hr1G065120.1), ubiquitin-protein ligase 2 (HORVU5Hr1G033930.8), and PEPC3, had significant changes in phosphorylation level under Pi starvation and resupply; 26S protease regulatory subunit 6B homolog (HORVU2Hr1G014000.1), TPS (HORVU3Hr1G071570.3), HXXXD-type acyl-transferase family protein (HORVU4Hr1G000930.1), PEPC 1 (HORVU5Hr1G055350.3), and sucrose phosphate synthase (SPS) 1F (HORVU6Hr1G028330.1) had significant changes in phosphorylation level under Pi starvation; and ubiquitin-specific protease family C19-related protein (HORVU2Hr1G100660.2) had significantly changed phosphorylation level under Pi resupply (Supplementary Table 8). Some of these enzymes have been shown to be extensively involved in the response to low Pi stress Hu, 2016; Jiang et al., 2018).

### Characteristics of the Different Phosphorylation States of One Protein in Response to Different Treatments

In this study, seven DPPs showed specific phosphorylation characteristics in response to both Pi starvation and resupply (Supplementary Table 9). For example, the polyol/monosaccharide transporter 5 (HORVU1Hr1G052040.1) had seven different serine phosphorylation sites with significant down-phosphorylation, with the exception of DDEDYAS(1)DHGADDIEDNLNSPLISR. Lupus La protein homolog (HORVU3Hr1G037470.19) had three different phosphorylation sites, the phosphorylation of KT (0.707) T (0.293) PPPVAGEAAVMGAESWPALEEAR and KT (0.5) T (0.5) PPPVAGEAAVMGAESWPALEEAR significantly differed for P6/CK and R6/CK, and LSS(0.013)S(0.987)PHGIPT(0.078)GS(0.654)S(0.267)PIGSVPK was only identified under P48/CK and R48/CK. The result suggests that these specific phosphorylation characteristics of DPPs may play a positive role in response to changes in P.

### Protein–Protein Interaction Network of Phosphorylated Proteins

**Figure 8:**
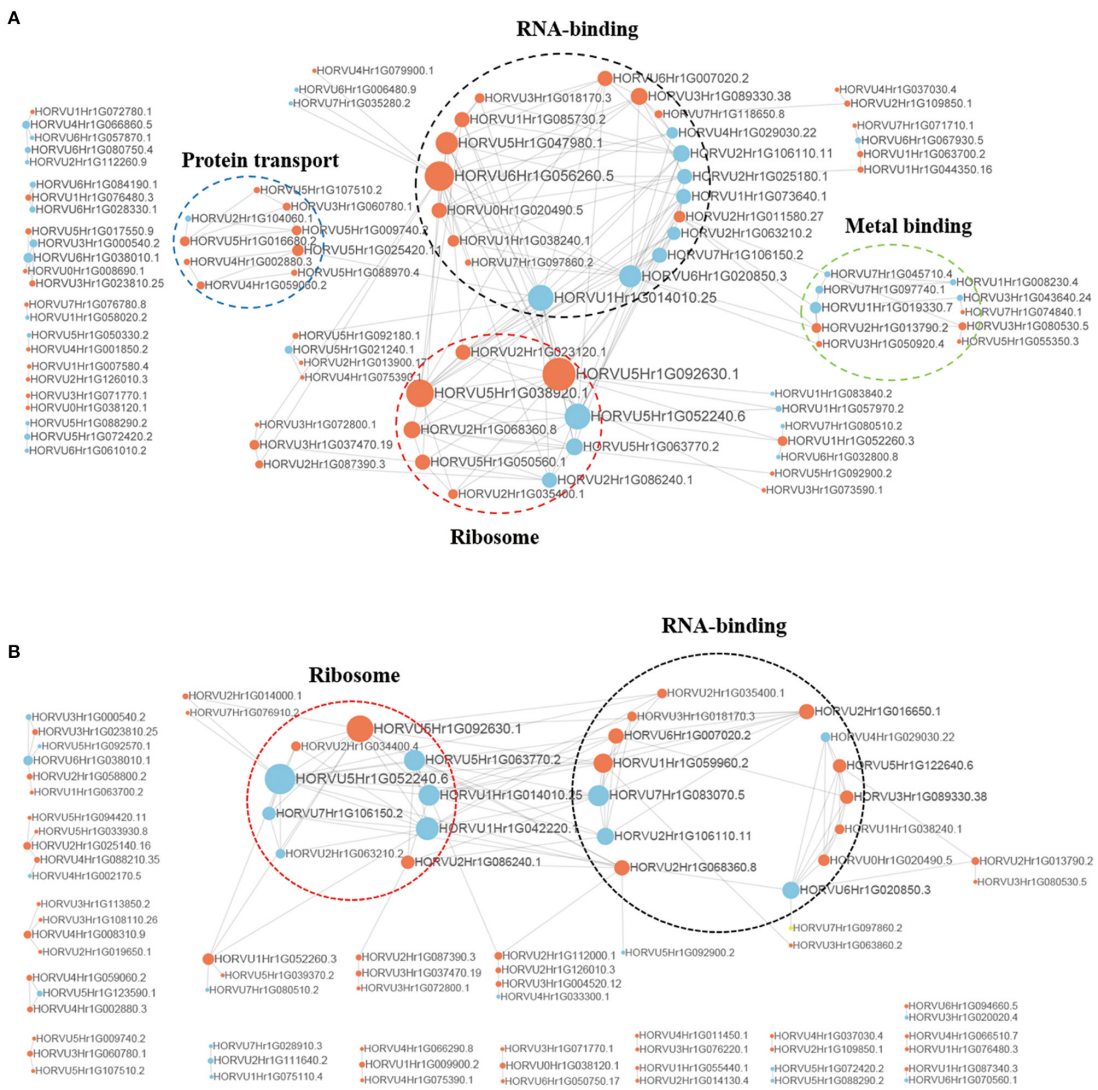
Interaction networks of phosphorylated proteins. **(A)** The whole PPI network of Pi starvation; **(B)** the whole PPI network of Pi resupply. Circle size represents the numbers of DPPs, and red indicates upregulated and blue indicates downregulated DPPs. DPPs, differentially phosphorylated proteins; PPI, protein–protein interaction.

To better understand the interactions between phosphorylated proteins, we created PPI for DPPs under Pi starvation and resupply stages, respectively (Figure 8 and Supplementary Table 10). To improve the reliability of PPI analysis, we retained all interactions that had a confidence score ≥0.7 (i.e., high confidence). The results showed that 94 DPPs were mapped to the protein interaction database under Pi starvation (Figure 8A). Of these, 56 were upregulated and 38 were downregulated, and seven DPPs were identified with node degree ≥20, including 60S ribosomal protein L5-1 (HORVU5Hr1G092630.1), nuclear cap-binding protein subunit 2(HORVU6Hr1G056260.5), and splicing factor 3B subunit 1 (HORVU6Hr1G020850.3). In general, the proteins were enriched for the pathway terms: RNA binding, ribosome, protein transport, and metal binding. For the Pi resupply stage, there were 84 DPPs as nodes, of which 59 were upregulated and 25 were downregulated, with the proteins mainly enriched in RNA binding and ribosome terms (Figure 8B). The phosphorylated proteins clearly had close interactions in the RNA-binding and ribosome terms under Pi starvation/resupply, and there were more phosphorylated proteins and metabolic processes involved in interactions for the Pi starvation treatment.

## Discussion

Reversible protein phosphorylation plays key roles in response to stress signals and in intracellular coordinating responses (Hu et al., 2015). In plants, the root is the main organ absorbing Pi and perceiving external nutrient stimuli (Marchsner, 2012). Recently, evidence suggests that protein phosphorylation is extensively involved in P absorption and transport responses to Pi starvation/resupply (Mehta et al., 2020; Wang et al., 2020a,b). The present study identified 7,710 phosphorylation sites on 3,373 proteins in barley roots, of which 327 DPPs were identified that were involved in a variety of metabolic processes and biological processes, such as signaling transduction pathway, carbon fixation, and carbohydrate metabolism pathway. The PPI network analysis also indicated that a wide range of interactions was regulated by phosphorylation.

### Phosphoproteins Involved in Phytohormone Signaling Pathway

Many phytohormones are involved in P signaling (Rubio et al., 2009; Wendrich et al., 2020). ABA plays a pivotal regulatory role in plant abiotic stress responses and can trigger major changes in plant physiology (Park et al., 2009; Raghavendra et al., 2010; de Zélicourt et al., 2016), mediated by the RCAR-PP2C-SnRK2 regulatory modules that are involved in reversible protein phosphorylation regulating processes (Zhang et al., 2018). The PP2Cs are key negative regulators of ABA-signaling pathways, which can inhibit the activity of SnRKs to cause the signaling cascade (Zhang et al., 2014a). In our study, two phosphatase 2C family proteins were identified, of which, HORVU1Hr1G093230.1 was significantly upregulated for 48 h of Pi starvation, indicating that the protein might actively participate in Pi tolerance by increasing phosphorylation. The HORVU7Hr1G049260.4 was significantly upregulated for 6 h under Pi starvation and resupply conditions, indicating that this PP2C protein had short-term activity for the P response. In addition, the KEGG pathway enrichment analysis showed that these PP2Cs were enriched in phytohormone signal transduction. The ABA receptors PYR/PYL/RCAR inhibited the activity of PP2Cs in an ABA-dependent manner (Rubio et al., 2009). The structure of the PYR1 monomer resembles a folded hand and completely wraps ABA in its central hydrophobic pocket by the interactions of polar and hydrophobic character to induce the PP2Cs to combine on the surface composed of gate and latch loops (Santiago et al., 2009). In the present study, we identified a PYR1(HORVU3Hr1G031380.1) protein that was downregulated at Ser219 under Pi starvation, but which was not identified under Pi resupply condition. We used the SWISS-MODEL server (https://swissmodel.expasy.org/) to predict the 3D protein structure models of PYR1, which showed that the phosphorylated site was present in the C-terminal part of helix α5 (Supplementary Figure 2); previous research showed that the Ser152 mutation caused a reduction in the stability of the ternary ABA–receptor–PP2C complex. Notably, Ser152 is located in the β7–α5 loop and does not contact the ABA molecule (Park et al., 2009). Thus, we speculate that the Ser219 phosphorylated in helix α5 of PYR1 had a positive function in the ABA signaling pathway.

In addition to ABA, ethylene took a key role in mediating adaptive responses to Pi stress that involved in the morphological changes of the root systems (Iqbal et al., 2013; Dubois et al., 2018). Roots treated with P-deficient stress generally produced more ethylene than P sufficient ones (Gilbert et al., 2000). EIN2 is the central positive regulator in the ethylene signaling pathway, which indicates CTR1 kinase-dependent dephosphorylation at several sites that were triggered by ethylene. Meanwhile, the EIN3/EIL1-dependent ethylene responses were stimulated by endoplasmic reticulum-nucleus translocation (Qiao et al., 2012). Most recently, the target of rapamycin (TOR) kinase has been reported to accurately regulate the phosphorylation of EIN2, and EIN2 protein plays an important role in regulating nutrient metabolism in plants under different nutrient levels and stress (Fu et al., 2021). In our research, the EIN2 (HORVU5Hr1G050330.2) was downregulated in the P6/CK at Ser269, but was upregulated in the R6/CK at Ser462. Moreover, the KEGG pathway enrichment analysis showed that EIN2 was enriched in the MAPK signaling pathway and plant hormone signal transduction. Hence, we speculate that the ethylene response to Pi starvation was triggered by the EIN2 dephosphorylation, but the ethylene reaction was prevented under the Pi resupply condition. Interestingly, phosphorylated EIN2 showed short-term activity at different phosphorylated sites in Pi starvation and resupply.

### Phosphoproteins Involved in the Carbohydrate Metabolism

In general, Pi that is absorbed by plant roots is esterified into hexose phosphate and ATP, which can provide energy to drive protein phosphorylation modification (Plaxton and Tran, 2011; Yang et al., 2019). However, P deficiency results in the promotion of glucose metabolism in plants, whereas the content of ATP decreases (Raghothama, 1999; Cai et al., 2012). Sucrose, a major regulator of plants, is transported from shoots to roots *via* the phloem in response to P starvation and is involved in morphological changes in roots, including lateral root and root hair development (Liu and Vance, 2010; Lei et al., 2011). However, sucrose synthesis is mainly catalyzed by SPS (Wu et al., 2014; Nemati et al., 2018), a plant-specific enzyme, with its activity controlled by a complex feedback mechanism, including phosphorylation/dephosphorylation (Wu et al., 2014). In this study, a SPS-1F (HORVU6Hr1G028330.1) was only identified in the Pi starvation treatment and showed a significantly downregulated phosphorylation level at Ser216, but with no significant difference after Pi resupply. Previous research showed that the phosphorylation sites of spinach and maize SPS were regulated by light/dark cycle and occurred at Ser158 and Ser162, respectively (McMichael et al., 1993; Takahashi et al., 2000). In addition, osmotic pressure also leads to significant changes of phosphorylation level in plant SPS (Toroser and Huber, 1997). Overall, the SPS was activated by dephosphorylation and inactivated by phosphorylation. Therefore, we suggest that SPS dephosphorylation may participate in the regulation of sucrose in barley under Pi starvation. Another example consistent with this phenomenon is TPS, a key enzyme in the trehalose biosynthetic process (Tischler et al., 2013). We identified four TPSs with significant changes in phosphorylation levels, among which HORVU3Hr1G071570.3 and HORVU6Hr1G084190.1 showed specific phosphorylation under Pi starvation condition, whereas HORVU4Hr1G066510.7 showed significantly upregulated specific phosphorylation under Pi resupply. In particular, the TPS (HORVU1Hr1G076480.3) showed phosphorylation at four sites (Ser5, Ser49, Ser70, and Ser71) under Pi starvation and showed a significant upregulation at Ser70 under Pi resupply. We speculate that these proteins were beneficial to the response of roots to P starvation by enhancing phosphorylation at more sites.

In the glycolysis pathway, PEPC is crucial, contributing to the plant tolerance of Pi starvation (Jiang et al., 2018). It catalyzes the metabolism of organic acids in roots to facilitate soil Pi solubilization in the rhizosphere, providing a glycolytic bypass (together with malate dehydrogenase and NAD-malic enzyme) to the ADP-limited cytosolic pyruvate kinase and Pi circulation of PEPC byproduct (Vance et al., 2003; Takahashi-Terada et al., 2005; Plaxton and Podestá, 2006; Chen et al., 2007). Previous studies showed that the serine residue of PEPC was specifically phosphorylated by PEPC kinase, for which the activity was allosterically controlled by second messengers, such as Ca^2+^ (Nimmo, 2003; Izui et al., 2004; Boxall et al., 2017). In this study, PEPC1(HORVU5Hr1G055350.3) showed significantly upregulated phosphorylation at Ser10 and Ser18 in the Pi starvation treatment. In support of our findings, PEPCs were researched to be phosphorylated in *A. thaliana* and *Lupinus albus* during Pi starvation (Uhde-Stone et al., 2003; Chen et al., 2007), and there was no detectable PEPC phosphorylation after Pi resupply (Gregory et al., 2009). In addition, we also identified a PEPC3 (HORVU7Hr1G080510.2) that showed downregulated phosphorylation at Ser13 for both Pi starvation and resupply. Previous studies found that plant PEPCs were activated by phosphorylation of the highly conserved Ser11 residue located near the N termini of PEPC1 and PEPC2, and the process reduced the sensitivity of PEPC to allosteric inhibitors, and increased its affinity to PEP (Li et al., 2020). Comparing PEPC sequences of barley with that in *A. thaliana* showed that two barley phosphorylated sites of Ser18 in PEPC1 and Ser13 in PEPC3 corresponded to the conserved site Ser11 in *A. thaliana* (Supplementary Figure 3). Notably, Ser10, a serine phosphorylation site, was identified for 6 h after Pi resupply in our research. There were three serine sites in PEPC1 and four serine sites in PEPC3 of barley that were close to the Ser11 site in *A. thaliana*. Thus, we speculate that these potential phosphorylation modification sites may play an active role at different stages of P starvation.

Fructose-bisphosphate aldolase is a key enzyme in glycolysis and gluconeogenesis. A previous study in drought-tolerant tomato showed that the gene encoding fructose-bisphosphate aldolase was downregulated by drought stress (Gong et al., 2010). In maize, drought, heat, and combined drought and heat stress resulted in increased phosphorylation levels of fructose-bisphosphate aldolase, but decreased protein expression (Hu et al., 2015). A proteomic study in rice roots showed that fructose-bisphosphate aldolase, phosphoglycerate mutase, and other related proteins related to glucose metabolism were upregulated by low Pi stress (Hu, 2016). In our study, we found that a fructose-bisphosphate aldolase 2 (HORVU3Hr1G088540.1) had downregulated phosphorylation for 6 h under Pi starvation. We speculated that reversible phosphorylation of fructose-bisphosphate aldolase might play an important role in plant adaptation to a wide range of abiotic stress, including Pi starvation.

### Phosphoproteins Involved in Signal Transduction

In numerous plant responses to abiotic stresses, Ca^2+^ is regarded as the second messenger in intracellular signaling (Davies, 2014). In P-deficient rice, Ca^2+^ concentration decreased in roots and increased in shoots and was possibly involved in regulating the Pi starvation response (Yang et al., 2019); in *Chlamydomonas reinhardtii*, two CDPKs (CDPK1 and CDPK3) were regulated by P and nitrogen starvation (Motiwalla et al., 2014). As Ca^2+^ sensors, CDPKs, calmodulin, and calceneurin B-like proteins play important and extensive roles in plant responses to several abiotic stresses (Luan et al., 2002; Hamel et al., 2014). Especially, CDPKs participate in various signal transduction pathways, such as ABA, drought, high salt, oxidative bursts, and osmotic stress (Motiwalla et al., 2014). In the current study, we identified two CDPKs that had significantly changed phosphorylation levels. In detail, CDPK33 (HORVU2Hr1G046660.6) showed upregulated phosphorylation in two sites (Ser13 and Ser32) for 6 h under the Pi starvation condition; CDPK19 (HORVU5Hr1G110900.3) showed increasing phosphorylation trends for 48 h after Pi starvation and resupply, indicating temporal specificity of phosphorylation. The CDPK family protein HORVU6Hr1G066470.4 showed upregulated phosphorylation level at Ser39 for 6 h under the Pi starvation condition, indicating that upregulated phosphorylation of some CDPKs might increase their activity in response to Pi starvation. However, calcium-dependent lipid-binding (CaLB) family protein HORVU2Hr1G036050.1 had downregulated phosphorylation at Ser474 under Pi starvation. In addition, we found that the phosphorylation level of calmodulin-binding transcription activator 1 (HORVU2Hr1G029780.1) and calmodulin-binding transcription activator 2 (HORVU4Hr1G078620.5) showed significantly upregulated phosphorylation at different serine phosphorylation sites under Pi starvation/resupply. These results indicate potential differences between periods and changes in P content in the phosphorylation modification of Pi homeostasis. As CaLB, annexins (such as AtANN1, OsANN1, OsANN3, and OsANN10) may be involved in Ca^2+^ channel regulation, peroxidase, and ATPase/GTPase activities in response to abiotic stress (Richards et al., 2013; Qiao et al., 2015; Li et al., 2019; Gao et al., 2020). In this study, annexin 4 (HORVU1Hr1G057170.11) had significantly increased phosphorylation levels under the Pi resupply condition. This indicates that annexin 4 may have a positive function in sensing changing signal responses to Pi resupply.

The ubiquitin proteasome system regulates PTM in plants and mainly participates in plant hormone responses and abiotic stress (Liu et al., 2020). However, phosphorylation usually functions as a tuning switch to govern ubiquitination pathways because of the lower energetic cost of a single kinase and ATP relative to ubiquitination, which has a higher energetic cost with its cascade of E1, E2, and E3 enzymes plus ubiquitin (Filipčík et al., 2017). In this study, we identified six ubiquitin-related proteins that had significantly changed phosphorylation level under Pi starvation/resupply, such as an ubiquitin family protein (HORVU1Hr1G052260.3), an ubiquitin-specific protease family C19-related protein (HORVU2Hr1G100660.2), ubiquitin-conjugating enzyme 23 (HORVU3Hr1G004520.12), ubiquitin carboxyl-terminal hydrolase 13 (HORVU3Hr1G075220.5), ubiquitin carboxyl-terminal hydrolase 26 (HORVU4Hr1G071690.11), and ubiquitin-protein ligase 2 (HORVU5Hr1G033930.8). In particular, ubiquitin-specific protease family C19-related protein and ubiquitin-conjugating enzyme 23 had significantly downregulated and upregulated phosphorylation under Pi resupply, respectively; and ubiquitin family protein (HORVU1Hr1G052260.3) phosphorylation was significantly upregulated at Ser31 at P48/CK, but downregulated at R48/CK. Previous studies have found that ubiquitin phosphorylation occurred on most eligible serine, threonine, and tyrosine residues (Swaney et al., 2013); the phosphorylation of Ser65 of ubiquitin, the phospho-ubiquitin event driving Parkinson's disease key enzymes of PINK1 or Parkin-Ubl is crucial to activate their activities and respond to mitochondrial damage (Ordureau et al., 2014; Pickrell and Youle, 2015). Thus, we speculate that these ubiquitin proteins may trigger a more sophisticated response to P starvation by regulating the phosphorylation level.

Many MAPK cascades are involved in plant responses to P (such as OsMAPK4, OsMAPK6, TaMPK6, and TaMPK1) (Wen et al., 2014; Yang et al., 2019), and the phosphorylation/dephosphorylation of the MAPK cascade is crucial for activation of enzymes that participate in transmitting and amplifying signals from specific environmental stimuli (Jonak et al., 1994). We found that a MAPK1 (HORVU7Hr1G097740.1) had downregulated phosphorylation at Ser193 under Pi resupply.

### Phosphoproteins Involved in Cell-Wall Stress

Primary cell walls consist of cellulose cross-linked by hemicelluloses or pectins (Tenhaken, 2015), and previous research has reported the involvement of the cell wall in plant responses to Pi starvation (Zhu et al., 2012). In this study, we found that three cellulose synthase-related proteins [cellulose synthase 1 (CESA1, HORVU0Hr1G038120.1) and cellulose synthase family proteins (HORVU3Hr1G071770.1 and HORVU6Hr1G050750.17)] had upregulated phosphorylation in response to Pi starvation/resupply. Compared with CK, the phosphorylation level of CESA1 showed at least nine quantification ratio changes at Ser226 for P6, P48, and R6. However, we find no significant phosphorylation for 48 h after Pi resupply, suggesting that phosphorylated CESA1 might play important role in the regulation of cellulose synthase in response to Pi starvation. In addition, the phosphorylation of CESA1 plays a critical role in regulating cell elongation, especially for rapidly expanding tissues (Chen et al., 2010). A cell-wall protein AWA1-like (HORVU1Hr1G089280.1) was also identified in P48/CK, with increased phosphorylation at Ser520. These results suggested that phosphorylation participated in cell-wall stress responses in barley roots under Pi starvation.

Many receptor-like kinase members have been found to perceive abiotic stress signals in the cell wall, and a large number of signaling proteins, including ABA, second messengers, reactive oxygen species (ROS), and phosphorylating transcription factors, are involved in the process of transmitting stress signals into the cell (Lindner et al., 2012; Wolf et al., 2012). We found that nine receptor kinases [receptor kinase 2 (HORVU4Hr1G015770.3, HORVU6Hr1G069980.1, and HORVU7Hr1G106450.1), receptor kinase 3 (HORVU2Hr1G042490.6, HORVU3Hr1G017020.13, and HORVU3Hr1G017020.13), receptor-like protein kinase 1 (HORVU4Hr1G019460.1 and HORVU7Hr1G055370.6), receptor-like kinase 902 (HORVU4Hr1G007550.1)] and a leucine-rich repeat receptor-like protein kinase family protein (HORVU4Hr1G066900.1) mostly showed upregulated phosphorylation under Pi starvation/resupply conditions.

### Phosphoproteins Involved in Oxidase Stress

The Plant NADPH oxidases participate in responses to various biotic and abiotic stresses by facilitating the production of ROS (Wang et al., 2018). In tomato under Pi starvation, the malondialdehyde and ROS levels increased to induce oxidative stress (Zhang et al., 2019b). Recently, a study demonstrated the close relationship between oxidative stress and root growth in barley (Wang et al., 2019). Maintaining root growth is a crucial strategy for plants to adapt to Pi starvation, which requires differential cell-wall synthesis and remodeling, including the activity of antioxidative enzymes (Tenhaken, 2015), such as peroxidase and glutathione S-transferase. We found that a peroxidase superfamily protein (HORVU5Hr1G037160.3) and a glutathione S-transferase family protein (HORVU1Hr1G017050.3) had downregulated phosphorylation under Pi resupply. This result indicated that the change in the phosphorylation level of the antioxidative enzymes may regulate their activity to maintain the ROS balance and protect plants from damage.

Respiratory burst oxidase homolog (*rboh*) genes can encode NADPH oxidase and have been identified in diverse plant species. In *A. thaliana*, a total of 10 *rboh* genes (*AtRbohA–J*) have been identified: *AtrbohB* plays a key role in the seed after-ripening; *AtRbohC, AtRbohH*, and *AtRbohJ* are essential for polar root hair growth (Mangano et al., 2017); and *AtrbohD* and *AtrbohF* mainly function in stress responses and phytohormone signaling (Torres et al., 2002; Kwak et al., 2003; Torres and Dangl, 2005; Desikan et al., 2006). It was reported that the phosphorylation of RbohF induced its activity and triggered the Ca^2+^-ROS signaling network (Kimura et al., 2012). In the present study, a respiratory burst oxidase protein F (HORVU3Hr1G087210.2) showed upregulated phosphorylation at Thr627 under Pi starvation, suggesting that phosphorylation could participate in ROS metabolism under Pi starvation.

### Model of the Phosphorylation Mechanisms Underlying Pi Starvation/Resupply in Barley Roots

**Figure 9:**
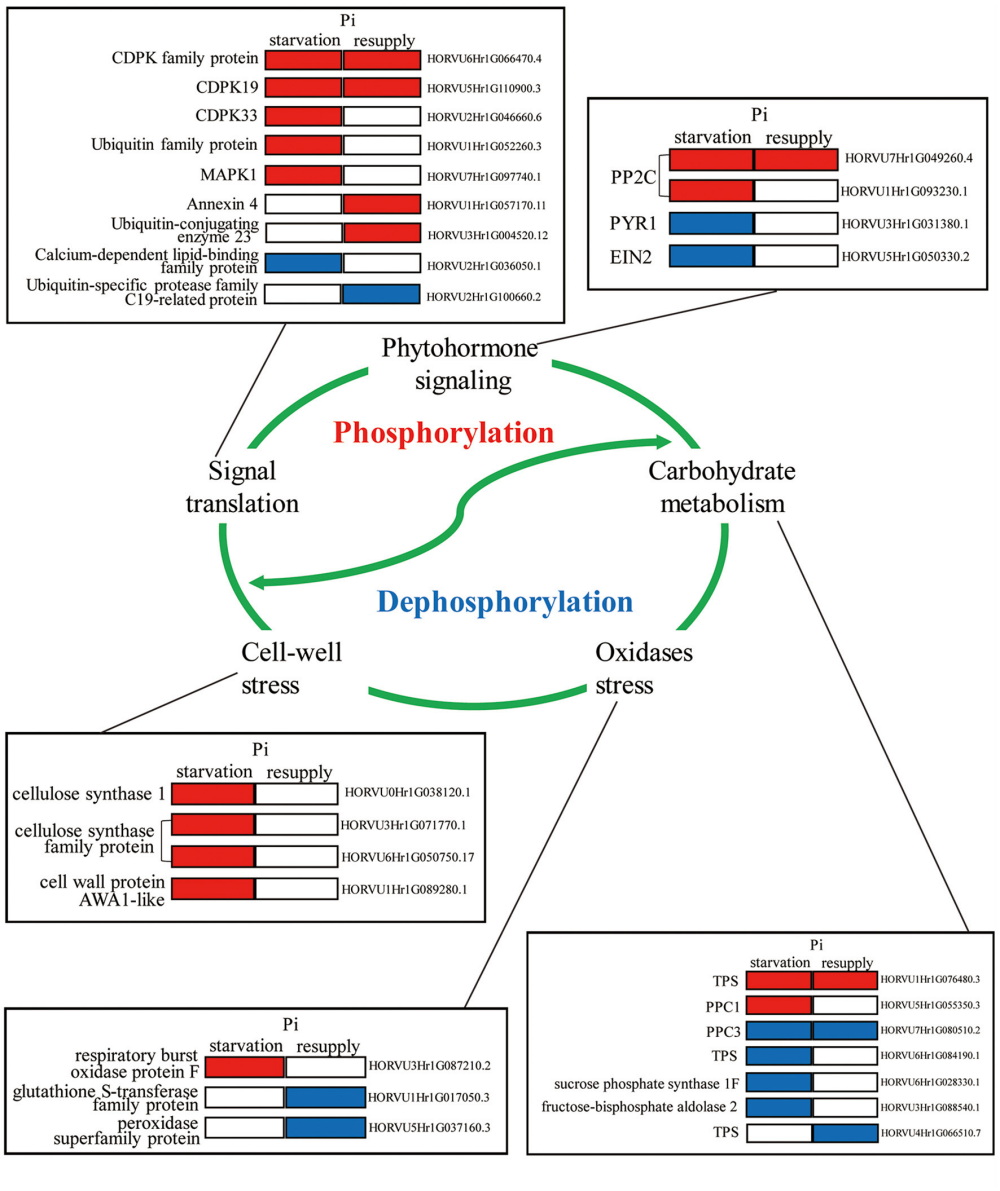
Molecular model of the underlying phosphorylation mechanisms involved in Pi starvation/resupply in barley roots.

To deeply research the biological processes of P response regulated by phosphorylation, we mainly analyzed the phosphorylated proteins involved in root growth and development under Pi starvation/resupply (Figure 9). We found that these phosphorylated proteins participated in diverse regulation of the root P responses, including phytohormone signaling, carbohydrate metabolism, signaling translation, cell-wall stress, and oxidases stress. For example, in phytohormone pathway, two phosphoproteins (PP2Cs) were upregulated and two phosphoproteins (PYR1 and EIN2) downregulated under Pi starvation condition. For signaling translation, most proteins were activated by phosphorylation under Pi starvation conditions, and Ca^2+^ had one of the most important roles. In particular, phosphoproteins related to cell-wall stress took an active role in the root Pi starvation responses. Additionally, these biological processes involved in the root P responses are linked by complex modes of action to perform their functions, and these need further exploration.

## Conclusion

This is the first extensive data on phosphorylation in barley roots under Pi starvation/resupply. A total of 7,710 phosphorylation sites corresponding to 3,373 proteins were identified. The phosphoproteins played key roles in diverse biological processes and metabolic pathways, especially in phytohormone signaling, carbohydrate metabolism, and Ca^2+^ signaling pathways, and the phosphoproteins involved in signaling pathways differed between Pi starvation and resupply. Our results not only broaden the range of metabolic processes known to be regulated by phosphorylation under Pi starvation/resupply conditions, but also provide new information for examining the functions of phosphorylation in barley roots. However, further studies are needed to reveal the effect of phosphorylation under Pi stress and to explain the potential mechanisms of response to Pi nutrition that are regulated by protein phosphorylation.

## Data Availability Statement

The original contributions presented in the study are publicly available. The mass spectrometry data have been deposited at the ProteomeXchange with dataset identifier PXD022053 and PXD022077.

## Author Contributions

ZM, JW, CL, and PR carried out the proteomic analysis and drafted the manuscript. LY, BL, YM, and XM participated in material culture and performed the statistical analysis. XS and HW conceived of the study and participated in its design. HW, ES, and KY helped to draft the manuscript. All authors read and approved the final manuscript.

## Conflict of Interest

The authors declare that the research was conducted in the absence of any commercial or financial relationships that could be construed as a potential conflict of interest.

## Supplementary Material

Supplementary Figure 1 The motifs of CDPKs, MAPKs and acidic kinases in barley roots under Pi starvation/resupply.

Supplementary Figure 2 The 3D protein structure models of PYR1 predicted by server SWISS-MODEL. The phosphorylation site Ser219 is shown in the dotted red circle.

Supplementary Figure 3 Sequence alignment of PPC1 and PPC3 from barley and *Arabidopsis thaliana*.

Supplementary Table 1 The identified phosphorylated sites in barley roots.

Supplementary Table 2 Motif statistics of phosphorylated peptides occurring on serine, threonine, and tyrosine.

Supplementary Table 3 Functional characterization and cellular localization of phosphorylated proteins.

Supplementary Table 4 Statistics of the differentially phosphorylated sites and proteins under Pi starvation for 6 h (P6/CK) and 48 h (P48/CK), and Pi resupply 6 h (R6/CK) and 48 h (R48/CK).

Supplementary Table 5 KEGG pathway enrichment analysis of DPPs in roots under Pi starvation for 6 h (P6/CK) and 48 h (P48/CK), and Pi resupply 6 h (R6/CK) and 48 h (R48/CK).

Supplementary Table 6 GO enrichment analysis of DPPs in roots under Pi starvation for 6 h (P6/CK) and 48 h (P48/CK), and Pi resupply 6 h (R6/CK) and 48 h (R48/CK).

Supplementary Table 7 InterPro domain enrichment of the DPPs in roots under Pi starvation for 6 h (P6/CK) and 48 h (P48/CK), and Pi resupply of 6 h (R6/CK) and 48 h (R48/CK).

Supplementary Table 8 Protein kinases, protein phosphatases, and other enzymes under Pi starvation at 6 h (P6/CK) and 48 h (P48/CK), and Pi resupply at 6 h (R6/CK) and 48 h (R48/CK).

Supplementary Table 9 Characteristics of several phosphopeptides belonging to one protein in response to Pi starvation (6 and 48 h) and resupply (6 and 48 h).

Supplementary Table 10 Overview of the phosphorylated proteins used to construct protein–protein interaction (PPI) network in response to Pi starvation and Pi resupply.

## References

[B1] Boxall S. Dever L. Knerova J. Gould P. Hartwell J. (2017). Phosphorylation of phosphoenolpyruvate carboxylase is essential for maximal and sustained dark CO_2_ fixation and core circadian clock operation in the obligate crassulacean acid metabolism species kalanchoë fedtschenkoi. Plant Cell 29:301. 10.1105/tpc.17.00301 28887405 PMC5774574

[B2] Cai J. Chen L. Qu H. Y. Lian J. Liu W. Hu Y. B.. (2012). Alteration of nutrient allocation and transporter genes expression in rice under N, P, K, and Mg deficiencies. Acta Physiol. Plant. 34, 939–946. 10.1007/s11738-011-0890-x

[B3] Chen S. Ehrhardt D. W. Somerville C. R. (2010). Mutations of cellulose synthase (cesa1) phosphorylation sites modulate anisotropic cell expansion and bidirectional mobility of cellulose synthase-mutations of cellulose synthase (cesa1) phosphorylation sites modulate anisotropic cell expansion and bidirectional mobility of cellulose synthase-supporting information. Proc. Natl. Acad. Sci. U.S.A. 107, 17188–17193. 10.1073/pnas.1012348107 20855602 PMC2951445

[B4] Chen Z. Nimmo G. A. Jenkins G. I. Nimmo H. G. (2007). BHLH32 modulates several biochemical and morphological processes that respond to Pi starvation in *Arabidopsis*. Biochem. J. 405, 191–198. 10.1042/BJ20070102 17376028 PMC1925254

[B5] Chiou T. J. Lin S. I. (2010). Signaling network in sensing phosphate availability in plants. Annu. Rev. Plant Biol. 62, 185–206. 10.1146/annurev-arplant-042110-103849 21370979

[B6] Cong W. F. Suriyagoda L. Lambers H. (2020). Tightening the phosphorus cycle through phosphorus-efficient crop genotypes. Trends Plant Sci. 25, 967–975. 10.1016/j.tplants.2020.04.013 32414603

[B7] Correl D. L. (1998). The role of phosphorus in the eutrophication of receiving waters. J. Environ. Qual. 27, 261–266. 10.2134/jeq1998.00472425002700020004x

[B8] Dai X. Wang Y. Zhang W. (2016). OsWRKY74, a WRKY transcription factor, modulates tolerance to phosphate starvation in rice. J. Exp. Bot. 67, 947–960. 10.1093/jxb/erv515 26663563 PMC4737085

[B9] Davies J. M. (2014). Annexin-mediated calcium signalling in plants. Plants 3, 128–140. 10.3390/plants3010128 27135495 PMC4844307

[B10] de Zélicourt A. Colcombet J. Hirt H. (2016). The role of MAPK modules and ABA during abiotic stress signaling. Trends in Plant Sci. 21, 677–685. 10.1016/j.tplants.2016.04.004 27143288

[B11] Desikan R. Last K. Harrett-Williams R. Tagliavia C. Harter K. Hooley R.. (2006). Ethylene-induced stomatal closure in *Arabidopsis* occurs via AtrbohF-mediated hydrogen peroxide synthesis. Plant J. 47, 907–916. 10.1111/j.1365-313X.2006.02842.x 16961732

[B12] Dubois M. Van den Broeck L. Inz,é D. (2018). The pivotal role of ethylene in plant growth. Trends Plant Sci. 23, 311–323. 10.1016/j.tplants.2018.01.003 29428350 PMC5890734

[B13] FAO. (2018). FAOSTAT Food and Agriculture Data. Available online at: http://www.fao.org/faostat/en/#home (accessed August 17, 2020).

[B14] Filipčík P. Curry J. R. Mace P. D. (2017). When worlds collide-mechanisms at the interface between phosphorylation and ubiquitination. J. Mol. Biol. 429, 1097–1113. 10.1016/j.jmb.2017.02.011 28235544

[B15] Fu L. Liu Y. Qin G. Wu P. Zi H. Xu Z.. (2021). The TOR-EIN2 axis mediates nuclear signalling to modulate plant growth. Nature 591, 1–5. 10.1038/s41586-021-03310-y 33658715

[B16] Gao S. Song T. Han J. He M. Zhang Q. Zhu Y.. (2020). A calcium-dependent lipid binding protein, OsANN10, is a negative regulator of osmotic stress tolerance in rice. Plant Sci. 293, 1104–1120. 10.1016/j.plantsci.2020.110420 32081268

[B17] Gilbert G. Knight J. Vance C. Allan D. (2000). Proteoid root development of phosphorus deficient lupin in mimicked by auxin and phosphonate. Ann. Bot. 85, 921–928. 10.1006/anbo.2000.1133

[B18] Gong P. Zhang J. Li H. Yang C. Zhang C. Zhang X.. (2010). Transcriptional profiles of drought-responsive genes in modulating transcription signal transduction, and biochemical pathways in tomato. J. Exp. Bot. 61, 3563–3575. 10.1093/jxb/erq167 20643807 PMC2921197

[B19] Gregory A. Hurley B. Tran H. Valentine A. She Y. M. Knowles V.. (2009). In vivo regulatory phosphorylation of the phosphoenolpyruvate carboxylase atppc1 in phosphate-starved *Arabidopsis thaliana*. Biochem. J. 420, 57–65. 10.1042/BJ20082397 19228119 PMC2677216

[B20] Hamel L. P. Sheen J. Seguin A. (2014). Ancient signals: comparative genomics of green plant CDPKs. Trends Plant Sci. 19, 79–89. 10.1016/j.tplants.2013.10.009 24342084 PMC3932502

[B21] Hou Y. Qiu J. Wang Y. Li Z. Zhao J. Tong X.. (2017). A quantitative proteomic analysis of brassinosteroid-induced protein phosphorylation in rice (*Oryza sativa* L.). Front. Plant Sci. 8:514. 10.3389/fpls.2017.00514 28439285 PMC5383725

[B22] Hu X. (2016). The discovery and verification of phosphorus efficient utilization related gene in rice roots. (Master's Thesis). Yangzhou University, China.

[B23] Hu X. Wu L. Zhao F. Zhang D. Li N. Zhu G.. (2015). Phosphoproteomic analysis of the response of maize leaves to drought, heat and their combination stress. Front. Plant Sci. 6:298. 10.3389/fpls.2015.00298 25999967 PMC4419667

[B24] Huang T. Han C. Lin S. Chen Y. Tsai Y. Chen Y.. (2013). Identification of downstream components of ubiquitin-conjugating enzyme PHOSPHATE2 by quantitative membrane proteomics in *Arabidopsis* roots. Plant Cell 25, 4044–4060. 10.1105/tpc.113.115998 24122829 PMC3877800

[B25] Iqbal N. Trivellini A. Masood A. Ferrante A. Khan N. A. (2013). Current understanding on ethylene signaling in plants: the influence of nutrient availability. Plant Physiol. Bioch. 73, 128–138. 10.1016/j.plaphy.2013.09.011 24095919

[B26] Izui K. Matsumura H. Furumoto T. Kai Y. (2004). Phosphoenolpyruvate carboxylase: a new era of structural biology. Annu. Rev. Plant Biol. 55, 69–84. 10.1146/annurev.arplant.55.031903.141619 15725057

[B27] Jiang J. Gai Z. Wang Y. Fan K. Sun L. Wang H.. (2018). Comprehensive proteome analyses of lysine acetylation in tea leaves by sensing nitrogen nutrition. BMC Genomics 19, 840. 10.1186/s12864-018-5250-4 30477445 PMC6258439

[B28] Johnston A. Poulton P. Fixen P. Curtin D. (2014). Phosphorus: its efficient use in agriculture. Adv. Agron. 123, 177–228. 10.1016/B978-0-12-420225-2.00005-4

[B29] Jonak C. Heberle-Bors E. Hirt H. (1994). MAP kinases: universal multi-purpose signaling tools. Plant Mol. Biol. 24, 407–416. 10.1007/BF00024109 8123784

[B30] Kafle A. Cope K. Raths R. Yakha J. Subramanian S. Bücking H.. (2019). Harnessing soil microbes to improve plant phosphate efficiency in cropping systems. Agronomy 9:127. 10.3390/agronomy9030127

[B31] Kimura S. Kaya H. Kawarazaki T. Hiraoka G. Senzaki E. Michikawa M.. (2012). Protein phosphorylation is a prerequisite for the Ca^2+^-dependent activation of *Arabidopsis* NADPH oxidases and may function as a trigger for the positive feedback regulation of Ca^2+^ and reactive oxygen species. Biochim. Biophys. Acta. 1823, 398–405. 10.1016/j.bbamcr.2011.09.011 22001402

[B32] Kwak J. Mori I. Pei Z. M. Leonhardt N. Torres M. A. Dangl J.. (2003). NADPH oxidase AtrbohD and AtrbohF genes function in ROS-dependent ABA signaling in Arabidopsis. EMBO J. 22. 2623–2633. 10.1093/emboj/cdg277 12773379 PMC156772

[B33] Lei M. Liu Y. Zhang B. Zhao Y. Wang X. Zhou Y.. (2011). Genetic and genomic evidence that sucrose is a global regulator of plant responses to phosphate starvation in *Arabidopsis*. Plant Physiol. 156, 1116–1130. 10.1104/pp.110.171736 21346170 PMC3135933

[B34] Li X. Sanagi M. Lu Y. Nomura Y. Stolze S. Yasuda S.. (2020). Protein phosphorylation dynamics under carbon/nitrogen-nutrient stress and identification of a cell death-related receptor-like kinase in *Arabidopsis*. Front. Plant Sci. 11:377. 10.3389/fpls.2020.00377 32308664 PMC7145971

[B35] Li X. Zhang Q. Yang X. Han J. Zhu Z. (2019). OsANN3, a calcium-dependent lipid binding annexin is a positive regulator of ABA-dependent stress tolerance in rice. Plant Sci. 284, 212–220. 10.1016/j.plantsci.2019.04.019 31084874

[B36] Lin W. Huang T. Chiou T. (2013). Nitrogen limitation adaptation, a target of microRNA827, mediates degradation of plasma membrane-localized phosphate transporters to maintain phosphate homeostasis in *Arabidopsis*. Plant Cell 25, 4061–4074. 10.1105/tpc.113.116012 24122828 PMC3877804

[B37] Lindner H. Mueller L. Boisson-Dernier A. Grossniklaus U. (2012). CrRLK1L receptor-like kinases: not just another brick in the wall. Curr. Opin. Plant Biol. 15, 659–669. 10.1016/j.pbi.2012.07.003 22884521

[B38] Liu J. Vance C. (2010). Crucial roles of sucrose and microrna399 in systemic signaling of p deficiency: a tale of two team players? Plant Signal. Behav. 5, 1556–1560. 10.4161/psb.5.12.13293 21139425 PMC3115102

[B39] Liu W. Tang X. Qi X. Fu X. Ghimire S. Ma R.. (2020). The ubiquitin conjugating enzyme: an important ubiquitin transfer platform in ubiquitin-proteasome system. Int. J. Mol. Sci. 21:2894. 10.3390/ijms21082894 32326224 PMC7215765

[B40] Lopez-Arredondo D. Leyva-Gonzalez M. Gonzalez-Morales S. Lopez-Bucio J. Herrera-Estrella L. (2014). Phosphate nutrition: improving low-phosphate tolerance in crops. Annu. Rev. Plant Biol. 65, 95–123. 10.1146/annurev-arplant-050213-035949 24579991

[B41] Luan S. Kudla J. Rodríguez-Concepción M. Yalovsky S. Gruissem W. (2002). Calmodulins and calcineurin B-like proteins: calcium sensors for specific signal response coupling in plants. Plant Cell 14, S389–400. 10.1105/tpc.001115 12045290 PMC151268

[B42] Luo B. Ma P. Nie Z. Zhang X. He X. Ding X.. (2018). Metabolite profiling and genome-wide association studies reveal response mechanisms of phosphorus deficiency in maize seedling. Plant J. 97, 947–969. 10.1111/tpj.14160 30472798 PMC6850195

[B43] Macdonald G. Bennett E. Potter P. Ramankutty N. (2011). Agronomic phosphorus imbalances across the world's croplands. Proc. Natl. Acad. Sci. U.S.A 108, 3086–3091. 10.1073/pnas.1010808108 21282605 PMC3041096

[B44] Mangano S. Denita-Juarez S. P. Choi H. S. Marzol E. Hwang Y. Ranocha P.. (2017). Molecular link between auxin and ROS-mediated polar growth. Proc. Natl. Acad. Sci. U.S.A. 114, 5289–5294. 10.1073/pnas.1701536114 28461488 PMC5441824

[B45] Marchsner P. (2012). Marschner's Mineral Nutrition of Higher Plants. Science Press

[B46] McMichael R. W. Klein R. Salvucci M. Huber S. (1993). Identification of the major regulatory phosphorylation site in sucrose-phosphate synthase. Arch. Biochem. Biophys. 307, 248–252. 10.1006/abbi.1993.1586 8274010

[B47] Mehta D. Ghahremani M. Pérez-Fernández M. Tan M. Schläpfer P. Plaxton W.. (2020). Phosphate and phosphite differentially impact the proteome and phosphoproteome of *Arabidopsis* suspension cell cultures. Plant J. 105, 924–941. 10.1111/tpj.15078 33184936

[B48] Motiwalla M. Sequeira M. D'Souza J. (2014). Two calcium-dependent protein kinases from chlamydomonas reinhardtii are transcriptionally regulated by nutrient starvation. Plant signal. Behav. 9:27969. 10.4161/psb.27969 24514873 PMC4091517

[B49] Nadira U. Ahmed I. Zeng J. Wu F. Zhang G. (2016). Identification of the differentially accumulated proteins associated with low phosphorus tolerance in a Tibetan wild barley accession. J. Plant Physiol. 198,10–22. 10.1016/j.jplph.2016.04.016 27111503

[B50] Nemati F. Ghanati F. Ahmadi G. H. Sharifi M. (2018). Comparison of sucrose metabolism in wheat seedlings during drought stress and subsequent recovery. Biol. Plant 62, 595–599. 10.1007/s10535-018-0792-5

[B51] Nimmo H. (2003). Control of the phosphorylation of phosphoenolpyruvate carboxylase in higher plants. Arch. Biochem. Biophys. 414, 189–196. 10.1016/S0003-9861(03)00115-2 12781770

[B52] Niu Y. Chai R. Jin G. Wang H. Tang C. Zhang Y. (2012). Responses of root architecture development to low phosphorus availability: a review. Ann. Bot. 112, 391–408. 10.1093/aob/mcs285 23267006 PMC3698383

[B53] Oldroyd G. Leyser O. (2020). A plant's diet, surviving in a variable nutrient environment. Science 368:eaba0196. 10.1126/science.aba0196 32241923

[B54] Ordureau A. Sarraf S. Duda D. Heo J. M. Jedrychowski M. Sviderskiy V.. (2014). Quantitative proteomics reveal a feedforward mechanism for mitochondrial PARKIN translocation and ubiquitin chain synthesis. Mol. Cell 56, 360–375. 10.1016/j.molcel.2014.09.007 25284222 PMC4254048

[B55] Park B. S. Seo J. S. Chua N. H. (2014). NITROGEN LIMITATION ADAPTATION recruits PHOSPHATE2 to target the phosphate transporter PT2 for degradation during the regulation of *Arabidopsis* phosphate homeostasis. Plant Cell 26, 454–464. 10.1105/tpc.113.120311 24474629 PMC3963589

[B56] Park S. Y. Fung P. Nishimura N. Jensen D. Fujii H. Zhao Y.. (2009). Abscisic acid inhibits type 2C protein phosphatases via the PYR/PYL family of START proteins. Science 324, 1068–1071. 10.1126/science.1173041 19407142 PMC2827199

[B57] Parra-Almuna L. Pontigo S. Larama G. Cumming J. Pérez-Tienda J. Ferrol N.. (2020). Expression analysis and functional characterization of two PHT1 family phosphate transporters in ryegrass. Planta 251:6. 10.1007/s00425-019-03313-0 31776735

[B58] Pickrell A. Youle R. (2015). The roles of PINK1, parkin, and mitochondrial fidelity in Parkinson's disease. Neuron 85, 257–273. 10.1016/j.neuron.2014.12.007 25611507 PMC4764997

[B59] Plaxton W. Podestá F. (2006). The functional organization and control of plant respiration. Crit. Rev. Plant Sci. 25, 15–198. 10.1080/07352680600563876

[B60] Plaxton W. Tran H. (2011). Metabolic adaptations of phosphate-starved plants. Plant Physiol. 156, 1006–1015. 10.1104/pp.111.175281 21562330 PMC3135920

[B61] Qiao B. Zhang Q. Liu D. Wang H. Yin J. Zhu Z.. (2015). A calcium-binding protein, rice annexin OsANN1, enhances heat stress tolerance by modulating the production of H_2_O_2_. J. Exp. Bot. 66, 5853–5866. 10.1093/jxb/erv294 26085678

[B62] Qiao H. Shen Z. Huang S. Schmitz R. Urich M. Briggs S.. (2012). Processing and subcellular trafficking of ER-tethered EIN2 control response to ethylene gas. Science 338, 390–393. 10.1126/science.1225974 22936567 PMC3523706

[B63] Raghavendra A. Gonugunta V. Christmann A. Grill E. (2010). ABA perception and signalling. Trends Plant Sci. 15, 395–401. 10.1016/j.tplants.2010.04.006 20493758

[B64] Raghothama K. (1999). Phosphate acquisition. Annu. Rev. Plant Biol. 50, 665–693. 10.1146/annurev.arplant.50.1.665 15012223

[B65] Reinders J. Sickmann A. (2005). State-of-the-art in phosphoproteomics. Proteomics 5, 4052–4061. 10.1002/pmic.200401289 16196093

[B66] Ren P. Ma X. Li B. Meng Y. Lai Y. Wang H.. (2016). Identification and selection of low-phosphate-tolerant germplasm in barley (*Hordeum vulgare* L.). Soil Sci. Plant Nutr. 62, 471–480. 10.1080/00380768.2016.1223521

[B67] Ren P. Meng Y. Li B. Ma X. Si E. Wang H.. (2018). Molecular mechanisms of acclimatization to phosphorus starvation and recovery underlying full-length transcriptome profiling in barley (*Hordeum vulgare* L.). Front. Plant Sci. 9:500. 10.3389/fpls.2018.00500 29720989 PMC5915550

[B68] Richards S. Laohavisit A. Mortimer J. Shabala L. Swarbreck S. Shabala S.. (2013). Annexin 1 regulates the H2O2-induced calcium signature in hero *Arabidopsis thaliana* roots. Plant J. 77, 136–145. 10.1111/tpj.12372 24180429

[B69] Rubio V. Bustos R. Irigoyen M. L. Cardona-López X. Rojas-Triana M. Paz-Ares J. (2009). Plant hormones and nutrient signaling. Plant Mol. Biol. 69, 361–373. 10.1007/s11103-008-9380-y 18688730

[B70] Santiago J. Dupeux F. Round A. Antoni R. Park S. Y. Jamin M.. (2009). The abscisic acid receptor pyr1 in complex with abscisic acid. Nature 462, 665–668. 10.1038/nature08591 19898494

[B71] Sawers R. Svane S. Quan C. Grønlund M. Wozniak B. Gebreselassie M. N.. (2017). Phosphorus acquisition efficiency in arbuscular mycorrhizal maize is correlated with the abundance of root-external hyphae and the accumulation of transcripts encoding PHT1 phosphate transporters. New Phytol. 214, 632–643. 10.1111/nph.14403 28098948

[B72] Sha A. Li M. Yang P. (2016). Identification of phosphorus deficiency responsive proteins in a high phosphorus acquisition soybean (*Glycine max*) cultivar through proteomic analysis. BBA- Proteins Proteom. 1864, 427–434. 10.1016/j.bbapap.2016.02.001 26853500

[B73] Swaney D. L. Beltrao P. Starita L. Guo A. Rush J. Fields S.. (2013). Global analysis of phosphorylation and ubiquitylation cross-talk in protein degradation. Nat. Methods 10, 676–682. 10.1038/nmeth.2519 23749301 PMC3868471

[B74] Takahashi S. Ono K. Ugaki M. Ishimaru K. Aoki N. Ohsugi R. (2000). Ser162-dependent inactivation of overproduced sucrose-phosphate synthase protein of maize leaf in transgenic rice plants. Plant Cell Physiol. 41, 977–981. 10.1093/pcp/pcd014 11038058

[B75] Takahashi-Terada A. Kotera M. Ohshima K. Furumoto T. Matsumura H. Kai Y.. (2005). Maize phosphoenolpyruvate carboxylase: mutations at the putative binding site for glucose 6-phosphate caused desensitization and abolished responsiveness to regulatory phosphorylation. J. Biol. Chem. 280, 11798–11806. 10.1074/jbc.M408768200 15665330

[B76] Tenhaken R. (2015). Cell wall remodeling under abiotic stress. Front. Plant Sci. 5:771. 10.3389/fpls.2014.00771 25709610 PMC4285730

[B77] Tischler D. Niescher S. Kaschabek S. Schlömann M. (2013). Trehalose phosphate synthases OtsA1 and OtsA2 of Rhodococcus opacus 1CP. Fems Microbiol. Letters 342, 113–122. 10.1111/1574-6968.12096 23398506

[B78] Toroser D. Huber S. (1997). Protein phosphorylation as a mechanism for osmotic-stress activation of sucrose-phosphate synthase in spinach leaves. Plant Physiol. 114, 947–955. 10.1104/pp.114.3.947 9232876 PMC158383

[B79] Torres M. A. Dangl J. (2005). Functions of the respiratory burst oxidase in biotic interactions, abiotic stress and development. Curr. Opin. Plant Biol. 8. 397–403. 10.1016/j.pbi.2005.05.014 15939662

[B80] Torres M. A. Dangl J. Jones J. (2002). *Arabidopsis* gp91phox homologues AtrbohD and AtrbohF are required for accumulation of reactive oxygen intermediates in the plant defense response. Proc. Natl. Acad. Sci. U.SA 99. 517–522. 10.1073/pnas.012452499 11756663 PMC117592

[B81] Uhde-Stone C. Gilbert G. Johnson J. Litjens R. Zinn K. Temple S.. (2003). Adaptation of white lupin to phosphorus deficiency involves enhanced expression of genes related to organic acid metabolism. Plant Soil 248, 99–116. 10.1023/A:1022335519879

[B82] Uhrig R. Labandera A. M. Moorhead G. (2013). *Arabidopsis* PPP family of serine/threonine protein phosphatases: many targets but few engines. Trends Plant Sci. 18, 505–513. 10.1016/j.tplants.2013.05.004 23790269

[B83] Vance C. Uhde-Stone C. Allan D. (2003). Phosphorus acquisition and use: critical adaptations by plants for securing a nonrenewable resource. New Phytol. 157, 427–447. 10.1046/j.1469-8137.2003.00695.x 33873400

[B84] Wang F. Cui P. Tian Y. Huang Y. Wang H. Liu F.. (2020a). Maize ZmPT7 regulates Pi uptake and redistribution which is modulated by phosphorylation. Plant Biotechnol. J. 18, 2406–2419. 10.1111/pbi.13414 32431055 PMC7680542

[B85] Wang F. Deng M. Chen J. He Q. Jia X. Guo H.. (2020b). CASEIN KINASE2-dependent phosphorylation of PHOSPHATE2 fine-tunes phosphate homeostasis in rice. Plant Physiol. 183, 250–262. 10.1104/pp.20.00078 32161109 PMC7210639

[B86] Wang H. Shabala L. Zhou M. Shabala S. (2019). Developing a high-throughput phenotyping method for oxidative stress tolerance in barley roots. Plant Methods 15. 10.1186/s13007-019-0397-9 30774702 PMC6364415

[B87] Wang J. Ma Z. Li C. Ren P. Yao L. Wang H.. (2021). Dynamic responses of barley root succinyl-proteome to short-term phosphate starvation and recovery. Front. Plant Sci. 12. 10.3389/fpls.2021.649147 33868348 PMC8045032

[B88] Wang W. Chen D. Zhang X. Cheng Y. Shen F. (2018). Role of plant respiratory burst oxidase homologs in stress responses. Free Radical Res. 52, 1–271. 10.1080/10715762.2018.1473572 29732902

[B89] Wen Y. Li X. Guo C. Ma C. Duan W. Lu W.. (2014). Characterization and expression analysis of mitogen-activated protein kinase cascade genes in wheat subjected to phosphorus and nitrogen deprivation, high salinity, and drought. J. Plant Biochem. Biot. 24, 184–196. 10.1007/s13562-014-0256-8

[B90] Wendrich J. Yang B. J. Vandamme N. Verstaen K. Smet W. Velde C.. (2020). Vascular transcription factors guide plant epidermal responses to limiting phosphate conditions. Science 370:aay4970. 10.1126/science.aay4970 32943451 PMC7116379

[B91] Wijk K. Friso G. Walther D. Schulze W. (2014). Meta-analysis of *Arabidopsis thaliana* phospho-proteomics data reveals compartmentalization of phosphorylation motifs. Plant Cell 26, 2367–2389. 10.1105/tpc.114.125815 24894044 PMC4114939

[B92] Wolf S. Hématy K. Höfte H. (2012). Growth control and cell wall signaling in plants. Ann. Rev. Plant Biol. 63, 381–407. 10.1146/annurev-arplant-042811-105449 22224451

[B93] Wu P. Shou H. Xu G. Lian X. (2013). Improvement of phosphorus efficiency in rice on the basis of understanding phosphate signaling and homeostasis. Curr. Opin. Plant Biol. 16, 205–212. 10.1016/j.pbi.2013.03.002 23566853

[B94] Wu X. Sklodowski K. Encke B. Schulze W. (2014). A kinase-phosphatase signaling module with BSK8 and BSL2 involved in regulation of sucrose-phosphate synthase. J. Proteome Res. 13, 3397–3409. 10.1021/pr5003164 24924143

[B95] Yang J. Xie M. Yang3 X. Liu B. Lin H. (2019). Phosphoproteomic profiling reveals the importance of CK2, MAPKs and CDPKs in response to phosphate starvation in rice. Plant Cell Physiol. 60, 2785–2796. 10.1093/pcp/pcz167 31424513

[B96] Yang Z. Yang J. Wang Y. Wang F. Mao W. He Q.. (2020). PROTEIN PHOSPHATASE95 regulates phosphate homeostasis by affecting phosphate transporter trafficking in rice. Plant Cell 32, 740–757. 10.1105/tpc.19.00685 31919298 PMC7054036

[B97] Zhang H. Huang L. Hong Y. Song F. (2016). BOTRYTIS-INDUCED KINASE1, a plasma membrane-localized receptor-like protein kinase, is a negative regulator of phosphate homeostasis in *Arabidopsis thaliana*. BMC Plant Biol. 16:152. 10.1186/s12870-016-0841-1 27389008 PMC4936243

[B98] Zhang L. Li X. Li D. Sun Y. Li Y. Luo Q.. (2018). Cark1 mediates aba signaling by phosphorylation of aba receptors. Cell Discov. 4:30. 10.1038/s41421-018-0029-y 29928509 PMC6006248

[B99] Zhang M. Lv D. Ge P. Bian Y. Chen G. Zhu G. (2014a). Phosphoproteome analysis reveals new drought response and defense mechanisms of seedling leaves in bread wheat (*Triticum aestivum* L.). J. Proteomics 109, 290–308. 10.1016/j.jprot.2014.07.010 25065648

[B100] Zhang M. Ma C. Lv D. Zhen S. Li X. Yan Y. (2014b). Comparative phosphoproteome analysis of the developing grains in bread wheat (*Triticum aestivum* L.) under well-watered and water-deficit conditions. J. Proteome Res. 13, 4281–4297. 10.1021/pr500400t 25145454

[B101] Zhang Y. Hu L. Yu D. Xu K. Zhang J. Li X.. (2019a). Integrative analysis of the wheat pht1 gene family reveals a novel member involved in arbuscular mycorrhizal phosphate transport and immunity. Cells 8:490. 10.3390/cells8050490 31121904 PMC6562588

[B102] Zhang Y. Liang Y. Zhao X. Jin X. Hou L. Shi Y.. (2019b). Silicon compensates phosphorus deficit-induced growth inhibition by improving photosynthetic capacity, antioxidant potential, and nutrient homeostasis in tomato. Agronomy 9:733. 10.3390/agronomy9110733

[B103] Zhong M. Li S. Huang F. Qiu J. Zhang J. Sheng Z.. (2017). The phosphoproteomic response of rice seedlings to cadmium stress. Int. J. Mol. Sci. 18:2055. 10.3390/ijms18102055 28953215 PMC5666737

[B104] Zhu X. Lei G. Jiang T. Liu Y. Li G. Zheng S. (2012). Cell wall polysaccharides are involved in p-deficiency-induced cd exclusion in *Arabidopsis thaliana*. Planta 236, 989–997. 10.1007/s00425-012-1652-8 22526505

