# Supplementary material for: Global Profiling of Phosphorylation Reveals the Barley Roots Response to Phosphorus Starvation and Resupply
Source: Front Plant Sci. 2021 Jul 14;12:676432. doi: 10.3389/fpls.2021.676432 (PMC8317692; doi:10.3389/fpls.2021.676432)
Supplement: Supplementary Figure 1 — The motifs of CDPKs, MAPKs and acidic kinases in barley roots under Pi starvation/resupply. [file Data_Sheet_1.zip › Supplementary Tables/Supplementary Table 9.docx]

Table S9 Characteristic of several phosphopeptides belonging to one protein in response to Pi starvation and resupply.

| **Protein name** | **Site** | **Sequence** | **Significant changes of phosphorylation level** | | | |
| --- | --- | --- | --- | --- | --- | --- |
|  |  |  | **P6/CK** | **P48/CK** | **R6/CK** | **R48/CK** |
| HORVU1Hr1G052040.1 | 267 | LYGAEEGVS(1)WIAR |  | 0.488 |  |  |
|  | 279 | GGGQS(1)ALGS(1)ALGLMSR | 0.547 | 0.628 |  | 0.642 |
|  | 283 | GGGQS(1)ALGS(1)ALGLMSR | 0.511 | 0.617 | 0.368 | 0.598 |
|  | 322 | MPEVMGS(1)MR | 0.633 |  |  |  |
|  | 336 | STLFPNFGSMFS(1)VAEQQQAK |  | 0.321 | 0.162 | 0.388 |
|  | 360 | DDEDYAS(1)DHGADDIEDNLNSPLISR |  | 1.504 |  |  |
|  | 503 | DLIEQQLAGPAMVHPS(1)EAVAK | 0.361 | 0.615 | 0.36 |  |
|  |  |  |  |  |  |  |
| HORVU1Hr1G059810.2 | 79 | AAAGSS(0.014)S(0.986)LPDLSSIYAAGGVAR | 2.213 |  | 2.276 |  |
|  | 177 | SPVASASTLESAEGS(1)DEDVVTK | 1.969 | 1.734 |  | 1.945 |
|  |  |  |  |  |  |  |
| HORVU1Hr1G076480.3 | 5 | S(0.999)YT(0.001)NLLDLAAGNFAALGPAGGGR | 2.013 |  |  |  |
|  | 49 | VMTVPGTLS(1)ELDDEDDER | 1.644 |  |  |  |
|  | 70 | AATSSVASDVPS(1)S(1)AICER | 2.997 | 2.531 | 2.314 | 3.988 |
|  | 71 | AATSSVASDVPS(1)S(1)AICER |  | 1.776 |  |  |
|  |  |  |  |  |  |  |
| HORVU2Hr1G014130.4 | 77 | LLPDGGDDS(0.983)DY(0.017)DREDGEEEDDGGGPPPHR | 1.66 |  |  |  |
|  | 133 | LGS(1)LVRPTTPK | 1.668 |  |  |  |
|  | (T)139 | LGSLVRPTT(1)PK | 2.211 | 1.553 | 1.82 | 1.518 |
|  |  |  |  |  |  |  |
| HORVU3Hr1G037470.19 | (T)39 | KT(0.707)T(0.293)PPPVAGEAAVMGAESWPALEEAR | 2.144 |  | 1.691 |  |
|  | (T)40 | KT(0.5)T(0.5)PPPVAGEAAVMGAESWPALEEAR | 2.167 |  | 1.709 |  |
|  | 756 | LSS(0.013)S(0.987)PHGIPT(0.078)GS(0.654)S(0.267)PIGSVPK |  | 1.649 |  | 2.076 |
|  |  |  |  |  |  |  |
| HORVU5Hr1G033930.8 | 1527 | DNS(1)PTQTSVVIDDSK |  |  | 0.414 |  |
|  | (T)2367 | RPMAEHPDGQS(0.104)T(0.896)PAAGAQVNDPPSNLHGPETDAR | 1.623 | 1.509 |  | 1.527 |
|  | 2693 | YHSGSLFGMNS(1)R | 1.569 |  | 1.587 |  |
|  |  |  |  |  |  |  |
| HORVU5Hr1G088290.2 | 342 | NMHNAS(1)DNGS(1)IRS(1)NRQDEDR | 0.236 | 0.479 | 0.368 | 0.442 |
|  | 346 | NMHNAS(1)DNGS(1)IRS(1)NRQDEDR | 0.256 | 0.52 | 0.363 | 0.48 |
|  | 349 | NMHNAS(1)DNGS(1)IRS(1)NRQDEDR |  | 0.592 | 0.487 | 0.579 |
