# Supplementary figures and images for: Global Profiling of Phosphorylation Reveals the Barley Roots Response to Phosphorus Starvation and Resupply
Source: Front Plant Sci. 2021 Jul 14;12:676432. doi: 10.3389/fpls.2021.676432 (PMC8317692; doi:10.3389/fpls.2021.676432)

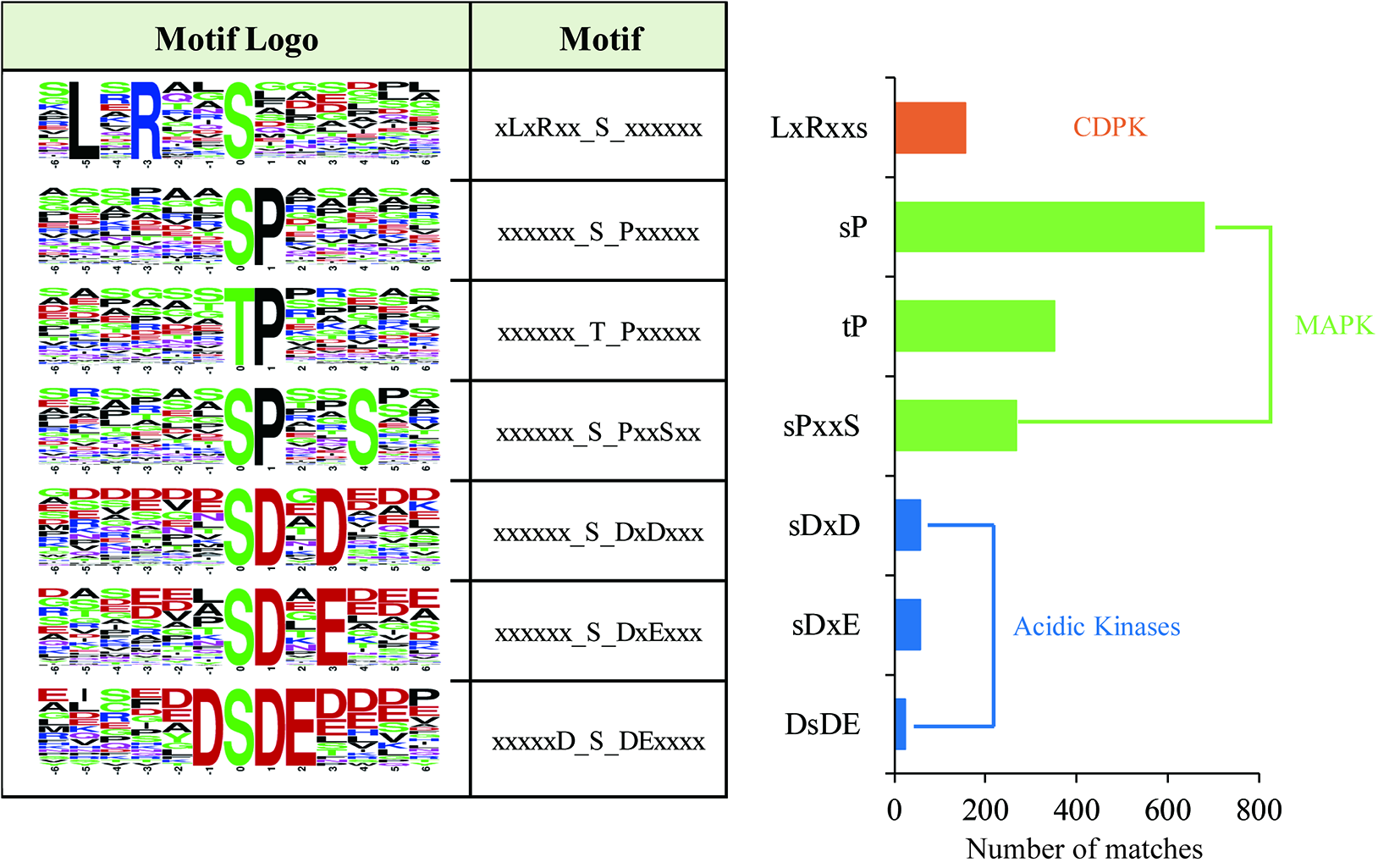

Supplement: Supplementary Figure 1 — The motifs of CDPKs, MAPKs and acidic kinases in barley roots under Pi starvation/resupply. [file Data_Sheet_1.zip › Supplmentary Figures/Supplementary Figure 1.tif]

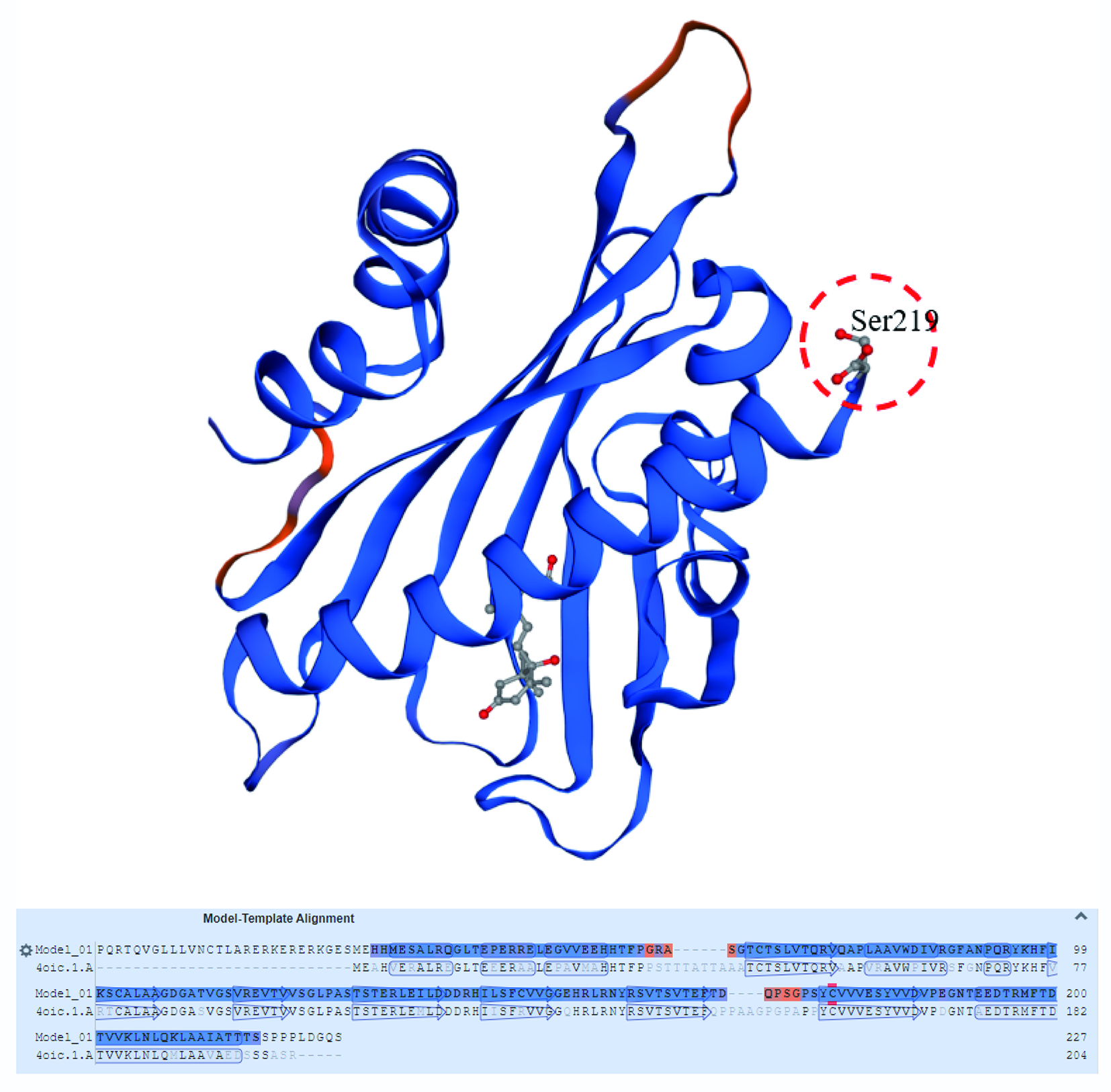

Supplement: Supplementary Figure 1 — The motifs of CDPKs, MAPKs and acidic kinases in barley roots under Pi starvation/resupply. [file Data_Sheet_1.zip › Supplmentary Figures/Supplementary Figure 2.tif]

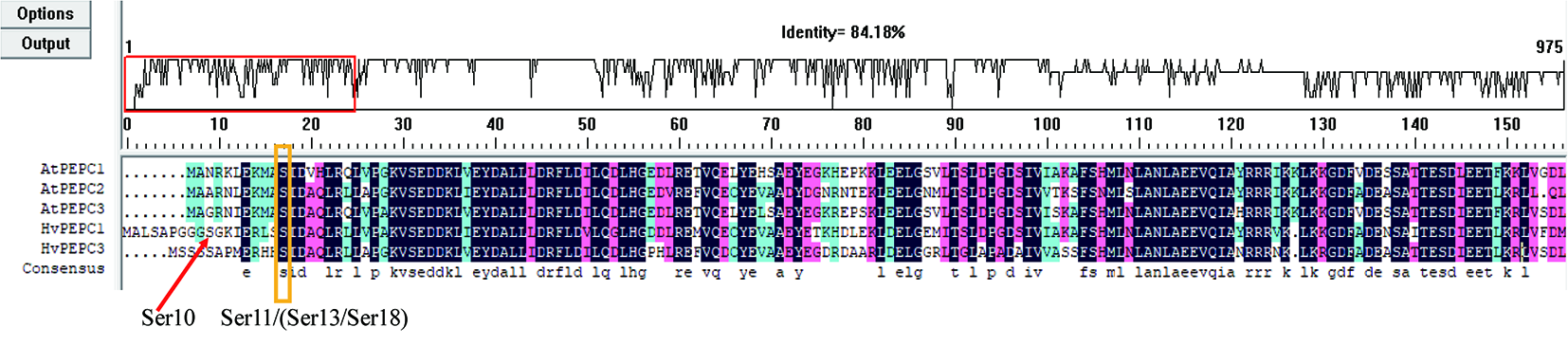

Supplement: Supplementary Figure 1 — The motifs of CDPKs, MAPKs and acidic kinases in barley roots under Pi starvation/resupply. [file Data_Sheet_1.zip › Supplmentary Figures/Supplementary Figure 3.tif]
